# Primary hypertension, anti-hypertensive medications and the risk of severe COVID-19 in UK Biobank

**DOI:** 10.1371/journal.pone.0276781

**Published:** 2022-11-09

**Authors:** Holly Pavey, Spoorthy Kulkarni, Angela Wood, Yoav Ben-Shlomo, Peter Sever, Carmel McEniery, Ian Wilkinson

**Affiliations:** 1 Division of Experimental Medicine and Immunotherapeutics, University of Cambridge, Cambridge, United Kingdom; 2 Department of Clinical Pharmacology, Cambridge University Hospitals NHS Foundation Trust, Cambridge, United Kingdom; 3 British Heart Foundation Cardiovascular Epidemiology Unit, Department of Public Health and Primary Care, University of Cambridge, Cambridge, United Kingdom; 4 British Heart Foundation Centre of Research Excellence, University of Cambridge, Cambridge, United Kingdom; 5 Health Data Research UK Cambridge, Wellcome Genome Campus and University of Cambridge, Cambridge, United Kingdom; 6 National Institute for Health Research Blood and Transplant Research Unit in Donor Health and Genomics, University of Cambridge, Cambridge, United Kingdom; 7 The Alan Turing Institute, London, United Kingdom; 8 Population Health Sciences, University of Bristol, Bristol, United Kingdom; 9 National Heart and Lung Institute, Imperial College London, London, United Kingdom; Cleveland Clinic Lerner Research Institute, UNITED STATES

## Abstract

Hypertension appears to be one of the commonest comorbidities in COVID-19 patients, although whether hypertensive individuals have a higher risk of severe COVID-19 compared with non-hypertensives is unclear. It is also unclear whether the absolute level of systolic blood pressure, or the type of anti-hypertensive medication is related to this risk. Analyses were conducted using data from the UK Biobank and linked health records. Logistic regression models were fitted to assess the impact of hypertension, systolic blood pressure (SBP) and medications on the risk of severe COVID-19. 16,134 individuals tested positive for severe acute respiratory syndrome-coronavirus, 22% (n = 3,584) developed severe COVID-19 and 40% (n = 6,517) were hypertensive. Hypertension was associated with 22% higher odds of severe COVID-19 (Odds ratio (OR) 1.22; 95% confidence interval (CI) 1.12, 1.33), compared with normotension after adjusting for confounding variables. In those taking anti-hypertensive medications, elevated SBP showed a dose-response relationship with severe COVID-19 (150-159mmHg versus 120-129mmHg (OR 1.91; 95% CI 1.44, 2.53), >180+mmHg versus 120-129mmHg (OR 1.93; 95% CI 1.06, 3.51)). SBP <120mmHg was associated with greater odds of severe COVID-19 (OR 1.40; 95% CI 1.11, 1.78). Angiotensin-converting enzyme inhibitors or angiotensin-II receptor blockers were not associated with altered risk of severe COVID-19. Hypertension is an important risk factor for COVID-19. A better understanding of the underlying mechanisms is warranted in case of more severe strains or other viruses in the future.

## Introduction

A number of studies have reported that hypertension is one of the most common comorbidities amongst individuals with COVID-19. Moreover, the risk of developing severe COVID-19 is increased in hypertensive individuals compared with normotensives, possibly due to enhanced Angiotensin-converting enzyme-2 (ACE-2) receptor expression [1–8]. Indeed, a meta-analysis and systematic review exploring COVID-19 and associated comorbidities, found that hypertension was more prevalent in severe and fatal cases (48%) compared with all cases of COVID-19 (25%) [9]. However, previous studies have not adjusted consistently for key confounding factors such as age, ethnicity and socioeconomic status, which are themselves predictors of hypertension and severe COVID-19 [1, 7, 9]. It also remains unclear whether, amongst hypertensive individuals, the risk of COVID-19 varies according to the level of systolic blood pressure (SBP) and/or type of antihypertensive medication. Many studies have now examined associations between Angiotensin-converting enzyme inhibitors (ACEi) or angiotensin-II receptor blockers (ARBs) and the risk of COVID-19, but not with a specific focus on hypertensive individuals. Ultimately, a better understanding of the relationship between hypertension, antihypertensives and severe COVID-19 may provide novel pathophysiological insights and help to reduce COVID-19 morbidity and mortality.

According to the 2018 Health Survey for England, 30% of males and 26% of females were living with hypertension in the UK, over 85% of whom were treated with blood pressure (BP) lowering medications [10]. Of particular relevance to the current COVID-19 pandemic are the use of antihypertensive agents acting directly on the renin-angiotensin-aldosterone system (RAAS), namely ACEi and ARBs. Theoretically, these agents may perturb the balance between angiotensin II signalling and the counter-regulatory Angiotensin 1–7 and alter ACE2 expression, which the SARS-COV2 virus binds to for cell entry [11]. Such effects may alter the susceptibility to severe COVID-19, perhaps by modulating the risk of lung injury [3, 12]. This led to uncertainty about which antihypertensives should be prescribed during the COVID-19 pandemic and a need for clinical evidence to aid guidance [3, 13].

Two recent meta-analyses found no evidence of any effect of ACEi or ARBs on a range of COVID-19 outcomes, including severe disease and concluded that drugs targeting the RAAS should continue to be used [14, 15]. Moreover, in observational studies focusing on hypertensive populations, no evidence was found of any worsening effect of ACEi or ARBs on outcomes of COVID-19, and that they may even be protective. However, the majority of these studies compared individuals taking ACEi/ARBs with those not on any antihypertensive medications [16–20]. Nevertheless, the BRACE-CORONA trial, which was the first randomised controlled trial examining the use of ACEi and ARBs in COVID-19 patients concluded that cardiac patients hospitalised with COVID-19 can safely continue taking these drugs [21]. The aim of this study was to estimate the risk of severe COVID-19 between individuals with and without hypertension and explore whether any increased risk of severe COVID-19 was dependent on the absolute BP and/or type of antihypertensive treatment.

## Methods

### Population and study design

Participants from the UK Biobank, a large population-based cohort study, with linked primary health care records, death records and COVID-19 test results were included in these analyses. We used 04/04/2021 as the censor date for all records and test results used for these analyses. The Strengthening the Reporting of Observational Studies in Epidemiology (STROBE) recommendations were used to guide the reporting of this analysis and the STROBE table made publically available.

### UK Biobank database

The UK Biobank is a longitudinal prospective study, initiated in 2006 and has recruited over 500,000 men and women aged between 40 and 69 years from the UK. It was established to investigate diseases in middle and older ages. At baseline (2006–2010) and over two further follow-up visits, a vast array of data, including physical measures, lifestyle data and biological samples, were collected. The UK Biobank additionally released primary care records for approximately 409,000 individuals for use solely in COVID-19 research, providing a wealth of further and longer-term health outcomes. This study was covered by the generic ethical approval for UK Biobank studies from the NHS National Research Ethics Service (Ref 11/NW/0382). This project is performed under project number 45885 and full written ethical approval has been granted via the UK Biobank.

### Definitions

For the main analyses, hypertension status was determined using primary and secondary ICD10 codes for essential (primary) hypertension (I10) obtained from the UK Biobank cohort data including self-reported data, hospital episodes from Hospital Episode Statistics (HES) data as well as primary health care records for the individuals, where available. Read coded clinical terms (version 2 (V2) and clinical terms version 3 (CTV3), Systematised Nomenclature of Medicine–Clinical Terms (SNOMED—CT), The Phoenix Partnership (TPP) and TPP local codes via SystmOne and Egton Medical Information Systems (EMIS) local codes were mapped to ICD10 codes for linked health records where ICD10 codes were not provided. An individual was defined as taking BP lowering medication if they were prescribed BP lowering medications on their most recent prescription record. SBP, diastolic BP (DBP) and body mass index (BMI) were obtained from the primary care clinical records (see data supplement for code lists), where available, otherwise from the UK Biobank database, taking the BP reading closest to the analysis baseline (16/03/2020). Current smokers were those with a record of being a current smoker at their most recent UK Biobank visit. The Townsend deprivation index (TDI) calculated immediately prior to the UK Biobank baseline visit was used as a measure of socio-economic status (SES). Each individual was assigned a score corresponding to the output area in which their postcode was located, based on the national census output areas. The TDI consisted of z-scores calculated from the deprivation scores; a greater deprivation index represents a higher level of deprivation. Ethnicity was dichotomised into white and non-white (due to small numbers) and sex was taken from the baseline UK Biobank visit. All data were assumed to be true at the analysis baseline. Comorbidity was defined as any of the following: peripheral vascular disease (PVD), myocardial infarction (MI), coronary heart disease (CHD) (excluding hypertension), angina, heart failure (HF) and arrhythmia or stroke at any one of the UK Biobank follow-up visits, prevalent at baseline or history of a CV comorbidity based on International Classification of Diseases 10 (ICD10) codes from hospital inpatient episodes or from primary health care records using primary or secondary positions of the diagnostic codes. Death due to COVID-19 was obtained using ICD10 codes from the death records, primary care records and hospital inpatient records. Further information including ICD10 code lists is included in the supplement.

Medications were obtained from the UK Biobank database, defined using British National Formulary (BNF) codes and from primary care records using dictionary of medicines and devices (dm+d) codes and local healthcare codes (TPP/EMIS). Relevant medications included ACEi, ARBs and other anti-hypertensives, as well as statins, which are commonly taken in combination with anti-hypertensive medications. All antihypertensive medications not defined as ACEi or ARBs were defined as ‘other’. All medication lists were checked by a clinician. Further information on specific medication lists included in these analyses and code lists can be found in the supplement.

### COVID-19 data

From March 16^th^ 2020, COVID-19 test results from SARS-COV2 polymerase chain reaction (PCR)-based swab tests, provided by Public Health England (PHE), were available to be linked to UK Biobank data. Our outcome of interest was severe COVID-19, defined as a positive test result requested from an in-patient unit (hospitalisation) or a COVID-19 related cause of death based on primary ICD10 codes: U071 and U072, since 16 March 2020 from the PHE lab tests and HES data. On the World Health Organisation (WHO) clinical progression scale this was defined as score between 4 and 10 (hospitalised: moderate disease, hospitalised: severe disease or dead) [22]. We also considered COVID-19 related cause of death as a sensitivity analysis. Individuals known to have been lost to follow up or who died prior to 16 March 2020 (initiation date of COVID-19 testing in UK Biobank) were excluded from the analyses. To minimise any misclassification bias and bias due to shielding, analyses were performed only on individuals who had a confirmed positive test result for or were diagnosed with COVID-19, making the assumption that all included participants had been exposed to the SARS-COV2 virus. Positive COVID-19 results since 16 March 2020 were additionally taken from linked primary care records.

### Statistical methods

Logistic regression was used to assess the association between hypertension, SBP or antihypertensive medication and the odds of severe COVID-19. All analyses were adjusted for age, sex, BMI, ethnicity, smoking status, diabetes status, SES and inflammation (C-reactive protein (CRP)) as these were proposed as potential confounders. To assess the direct effect of hypertension on COVID-19, we adjusted for intermediate variables on the causal pathway between hypertension and severe COVID-19, these included CV comorbidities and Stroke (S20 Table in S1 File). These variables were additionally adjusted for when modelling SBP and type of antihypertensive medications. The linearity of continuous variables was assessed and all two-way interactions were considered and included only if the coefficient of the independent variable was altered significantly. Since the relationship between SBP and severe COVID-19 was non-linear, SBP and other continuous variables were categorised for ease of interpretation by the reader. Age was categorised in to decades: <60, ≥60 to <70, ≥70 to <80 and ≥80 years. BMI was categorised based on general BMI classifications: <25, ≥25 to <30, ≥30 to <35 and ≥35 kg/m^2^, classified as normal, overweight, class 1 obesity and class 2 obesity, respectively. SBP was categorised by 10mmHg ranges, starting from <120mmHg up to 180+mmHg, with the reference category defined as: 120-129mmHg, based on data from the SPRINT study demonstrating that intensive SBP lowering to below 120mmHg, as compared with the traditional threshold of 140mmHg, is beneficial [23]. DBP was categorised by 10mmHg ranges, starting from <60mmHg up to 100+mmHg with 80-90mmHg being the reference category. CRP was categorised into four categories: <3 mg/L, ≥3 to <10 mg/L, ≥10 to <100 mg/L and 100+ mg/L, classified as normal, minor elevations, moderately elevated and elevated. Analyses were performed on the subset of individuals with complete cases for the required variables.

Since logistic regression assumes that the underlying rate of COVID-19 was uniform across the analysis period, we repeated the key analyses using a cox proportional hazard model, with time to severe COVID-19 as the outcome. This allowed for variations in the underlying rate of COVID-19 infection over time. A number of sensitivity analyses were then considered including: (i) broadening our classification of hypertension to additionally include any individual whose most recent BP measurement indicated SBP ≥140mmHg or DBP ≥90mmHg (ii) assessing the association between hypertension with death due to COVID-19 as the outcome, (iii) considering the effect of SBP on the odds of severe COVID-19 in both treated and untreated individuals with hypertension, (iv) using the average SBP since 2016, (v) considering treatment by mono- or combination therapy, as well as considering individuals who had not switched between ACEi and ARB treatment. We also looked at the association between DBP and severe COVID-19. When comparing ACEi and ARB treatments with other antihypertensive medications, individuals reported to have been treated with both ACEi and ARB drug classes concurrently in their most recent prescription were removed from the analyses. Rv4.0.0 for RStudio was used to perform all analyses.

## Results

**Fig 1:**
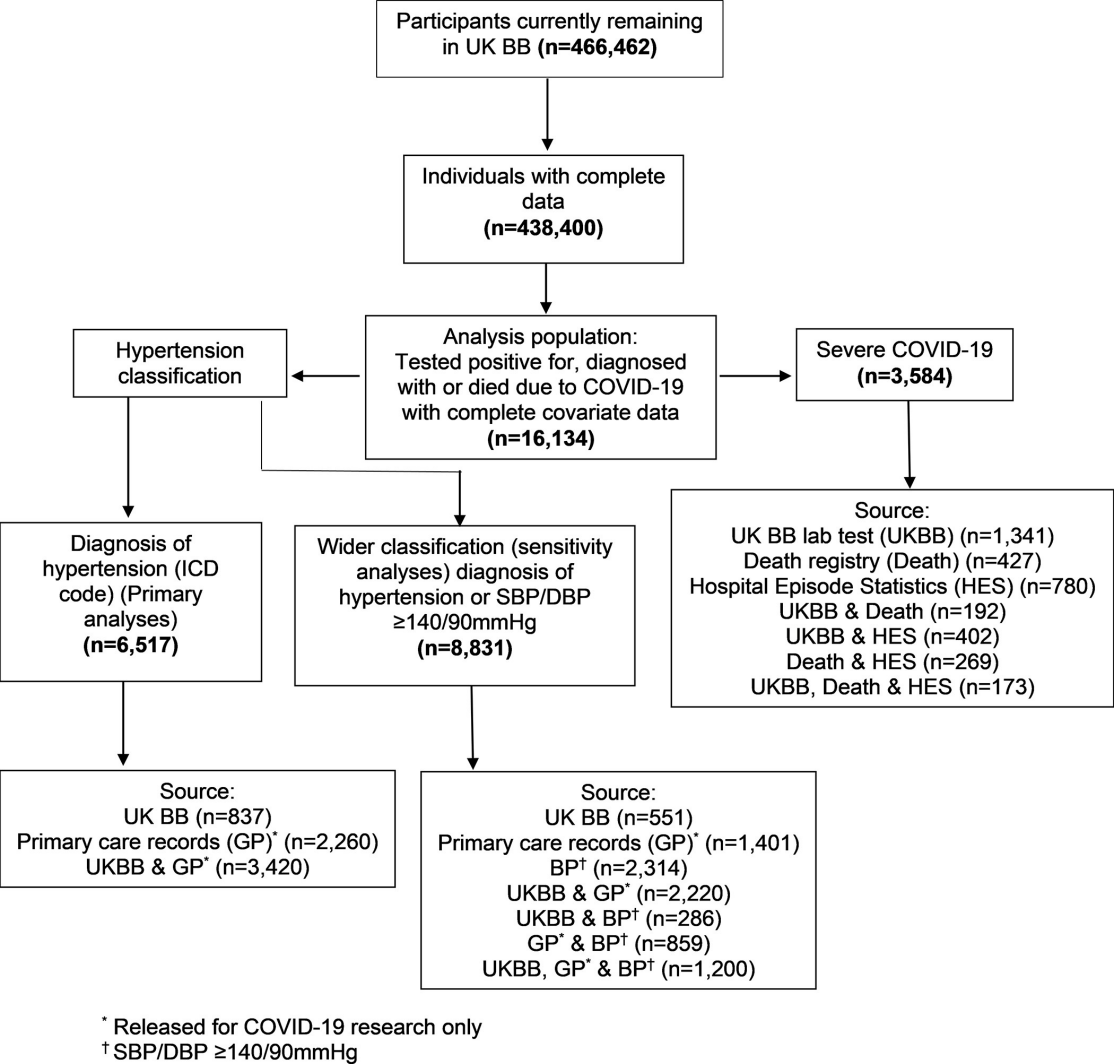
Flow chart to show the population derivation.

In all, 16,134 individuals who tested positive for, who were diagnosed with or who died due to COVID-19, with complete covariate data, were included in the analyses (Fig 1)). The characteristics of the cohort are presented in Table 1. The mean (SD) age was 65.3 (8.7) years, 47% were male, 90% were white and 40% were diagnosed with essential hypertension at the analysis baseline. Of the 16,134 individuals included in the analyses, SBP values were obtained from primary care records for 14,253 (88%) individuals. Severe COVID-19 occurred in 3,584 (22%) individuals, of whom 1,061 (30%) died. Individuals with severe COVID-19 were marginally older, more likely to be male (57%) and more deprived. They were also more likely to be hypertensive (56%, n = 2,024) compared with individuals without severe COVID-19 (36%, n = 4,493) and a greater proportion of individuals with severe COVID-19 had CV comorbidities.

**Table 1:** Baseline characteristics (N = 16,134).

Variable		No Severe Covid (N = 12,550)	Severe Covid (N = 3,584)	Total (N = 16,134)
**Age**	Mean (SD*)	63.8 (8.2)	70.5 (8.2)	65.3 (8.7)
**Sex: Male**	**n (%)** ^**†**^	5,619 (45%)	2,026 (57%)	7,645 (47%)
**SES**	**Median (Min, Max)**	-1.6 (-6.3, 10.6)	-1.2 (-6.3, 10.0)	-1.5 (-6.3, 10.6)
**Ethnicity: White**	**n (%)** ^**†**^	11,273 (90%)	3,274 (91%)	16,134 (90%)
**Non-White**	**n (%)** ^**†**^	1,277 (10%)	310 (9%)	1,587 (10%)
**BMI (kg/m** ^**2**^ **)**	**Mean (SD)**	27.9 (5.3)	29.1 (6.1)	28.2 (5.5)
**Current Smoker**	**n (%)** ^**†**^	1,354 (11%)	505 (14%)	1,859 (12%)
**SBP (mmHg)**	**Mean (SD)**	130 (15)	134 (16)	131 (15)
**DBP (mmHg)**	**Mean (SD)**	78 (9)	78 (11)	78 (10)
**PP (mmHg)**	**Mean (SD)**	53 (12)	56 (13)	53 (13)
**CRP**	**Median (Min, Max)**	1.52 (0, 353)	2.16 (0, 237)	1.65 (0, 353)
**Co-morbidities (n (%))** *				
**Diabetes**		1,437 (11%)	901 (25%)	2,338 (14%)
**PVD**		434 (3%)	268 (7%)	702 (4%)
**Arrhythmia**		1,205 (10%)	739 (21%)	1,944 (12%)
**Hypertensive HD** *		12 (0%)	12 (0%)	24 (0%)
**Angina**		866 (7%)	587 (16%)	1,453 (9%)
**MI**		373 (3%)	279 (8%)	652 (4%)
**Heart Failure**		254 (2%)	294 (8%)	548 (3%)
**Ischemic heart disease**		863 (7%)	635 (18%)	1,498 (9%)
**Stroke**		209 (2%)	182 (5%)	391 (2%)
**COPD** ^**†**^		449 (4%)	423 (12%)	872 (5%)
**Hypertension**^‡^ **n (%)**^**§**^				
**No hypertension**		8,057 (64%)	1,560 (44%)	9,617 (60%)
**Hypertension**	**(Treated)**	2,879 (23%)	1,512 (42%)	4,391 (27%)
	**(Untreated)**	1,614 (13%)	512 (14%)	2,126 (13%)

*SD = standard deviation

^†^ COPD = Chronic obstructive pulmonary disease

^‡^All percentages are column percentages

^**§**^ Chi-squared test of hypertension status (hypertension vs no hypertension) and severe COVID-19: chi-squared = 526.3, p-value <0.001

A total of 6,517 (40%) individuals had a diagnosis of essential hypertension, of whom 4,391 (67%) were treated (41% monotherapy (n = 1,798), 59% combination therapy (n = 2,593)) and 2,126 (33%) were untreated. The full breakdown of anti-hypertensive and other treatments is provided in Table 2. Twenty-nine (<0.01%) individuals were treated with both ACEi and ARBs, and were excluded from analyses considering the effect of antihypertensive medications on the odds of severe COVID-19. There were similar numbers of severe COVID-19 in each medication group: 34% (n = 685), 34% (n = 536) and 36% (n = 281) for ‘other’ medications, ACEi and ARBs, respectively. Antihypertensive medications were broadly comparable between those with and without severe COVID-19, but statins and anticoagulants were more prevalent amongst individuals with severe COVID-19. Fig 1 shows how the diagnoses of severe COVID-19 and hypertension were obtained.

**Table 2:** Antihypertensive medications, statins and anticoagulants taken by individuals with essential (primary) hypertension based on ICD10* code I10 (N = 6,899).

4	No Severe Covid (N = 4,493)	Severe Covid (N = 2,024)	Total (N = 6,517)
**ARB** ^**†**^	177 (4%)	80 (4%)	257 (4%)
**ACEi** ^‡^	469 (10%)	187 (9%)	656 (10%)
**ACEi + Other**	575 (13%)	349 (17%)	924 (14%)
**ARB + ACEi**	4 (0%)	2 (0%)	6 (0%)
**ARB + ACEi + Other**	15 (0%)	8 (0%)	23 (0%)
**ARB + Other**	320 (7%)	201 (10%)	521 (8%)
**Other**	1,319(29%)	685 (34%)	2,004 (31%)
**None**	1,614 (36%)	512 (25%)	2,126 (33%)
**Statins**	1,307 (29%)	831 (41%)	2,138 (33%)
**Anticoagulants**	183 (4%)	184 (9%)	367 (6%)

*ICD10 = international classification of disease (Tenth revision)

^**†**^ARB = angiotensin-receptor blockers

^‡^ACEi = angiotensin converting enzyme inhibitors

Statistics are displayed as n (%)

The unadjusted odds ratio (OR) of the association between hypertension and severe COVID-19 was 2.33 (95% CI: 2.16, 2.51); (Table 3). Adjusting for age greatly attenuated the association (age adjusted OR: 1.47 (1.35, 1.59)), as did diabetes (OR: 2.00 (1.85, 2.16)) and BMI (OR: 2.20 (2.04, 2.38)) (S1 Table in S1 File). After concurrently adjusting for all potential confounders, the adjusted OR was 1.22 (1.12, 1.33) (p<0.01) (Table 3 and S1 Table in S1 File). A sensitivity analysis revealed that the effect of hypertension on the hard outcome of death due to COVID-19 was similar to development of severe COVID-19 (OR: 1.17 (1.01, 1.36); S1 Table in S1 File). After further adjusting for possible effect mediators (CV comorbidities and stroke), there was a modest attenuation in the association between hypertension and severe COVID-19 (OR: 1.15 (1.05, 1.26); S2 Table in S1 File). Of note, those with a history of stroke had a 47% higher risk of severe COVID-19 (OR 1.47 (1.18, 1.83)) and those with a history of other CV comorbidities had a 30% higher risk of severe COVID-19 (OR: 1.30 (1.18, 1.43)). These results were similar when time to severe COVID-19 was analysed using a time-to-event model, over a 384 day follow-up period (S3 Table in S1 File).

**Table 3:** Association between hypertension and the odds of severe COVID-19 (N = 16,134).

Adjustment	Odds ratio	p-value
**Unadjusted**	2.33 (2.16, 2.51)	<0.001
**Age & Sex**	1.52 (1.40, 1.65)	<0.001
**Fully adjusted** ^*^	1.22 (1.12, 1.33)	<0.001
**Fully adjusted + CV comorbidities**^**†**^ **& stroke**	1.15 (1.05, 1.26)	<0.001

*Adjusted for sex, age, Townsend deprivation index, BMI, diabetes, smoking status, ethnicity and C-reactive protein

^**†**^ Not including stroke

**Fig 2:**
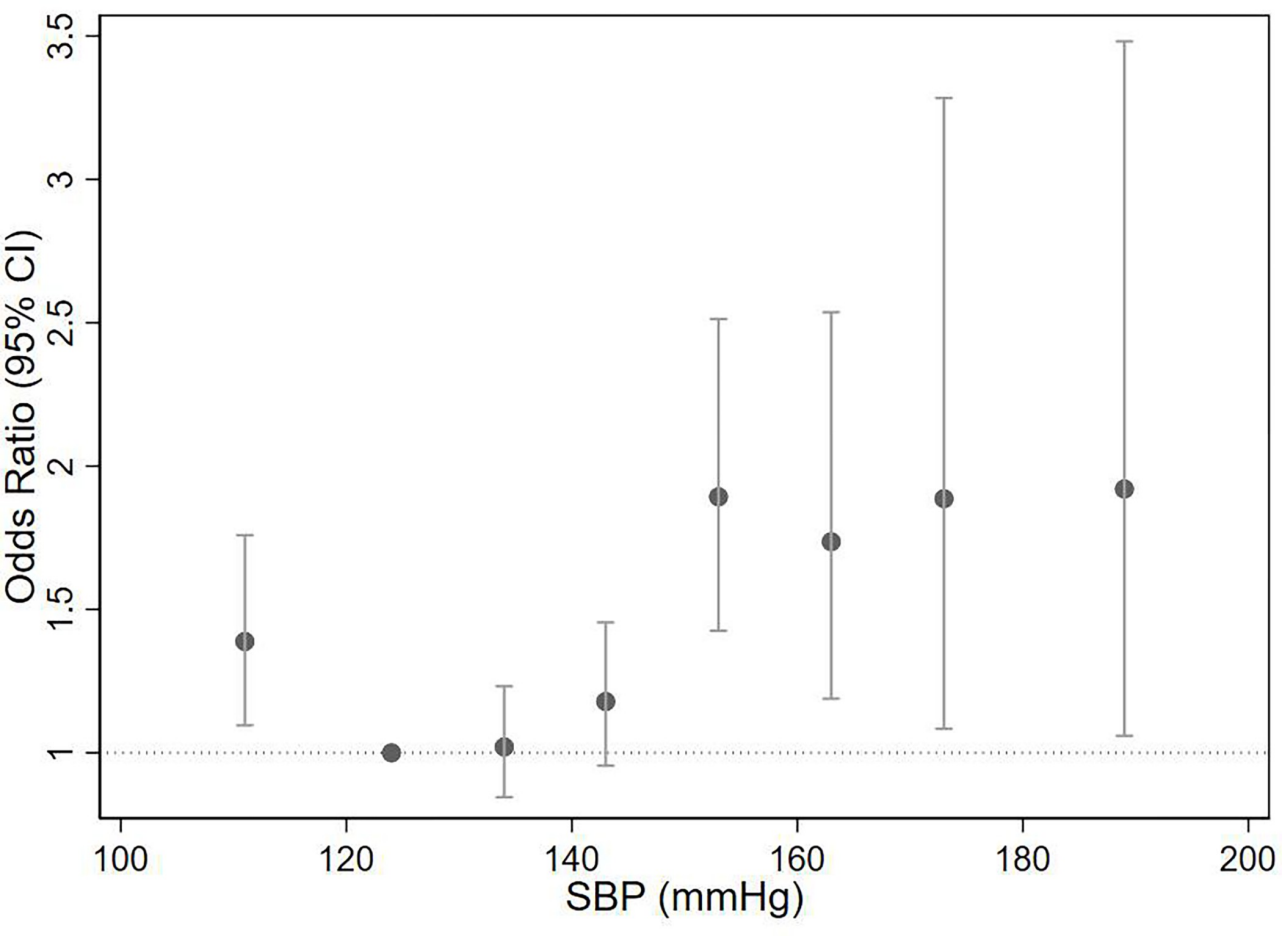
Odds ratio with 95% confidence interval for the fully adjusted model* for mean SBP within each category^†^ on the risk of severe COVID-19 in individuals with hypertension who are treated with antihypertensive medications. *Model adjusted for Townsend deprivation index (measure of socioeconomic status (SES)), diabetes, smoking, ethnicity, age group, BMI category, CV comorbidity, stroke and male sex. ^**†**^SBP was categorised by 10mmHg ranges, starting from <120mmHg up to 180+mmHg, with the reference category defined as: 120-129mmHg.

In hypertensive individuals receiving antihypertensive medications, there was a J-shaped relationship between the level of BP and risk of severe COVID-19, using a SBP level of 120–129 mmHg as a reference (Fig 2). A SBP of 150-159mmHg was associated with a 91% higher risk of suffering severe COVID-19 compared with a SBP of 120-129mmHg (OR: 1.91 (1.44, 2.53), p<0.001). There was no evidence of a higher risk of severe COVID-19 until SBP exceeded 150mmHg. Interestingly, a very well controlled or low BP (i.e. SBP<120mmHg) was associated with a 40% higher risk of severe COVID-19 compared with the reference BP (OR: 1.40 (1.11, 1.8), p = 0.005); S4 Table in S1 File). S5 Table in S1 File shows the results where SBP was modelled as a continuous variable, this association was non-linear. Modelling the time to severe COVID-19 showed comparable results (S15 Table in S1 File). The results were similar when restricted to individuals treated by combination therapy, but were less apparent when restricted to individuals taking monotherapy only and when averaging the SBP since 2016 (S6–S8 Tables in S1 File). Analyses including all hypertensive individuals further stratified by whether or not they were taking antihypertensive medications showed that there was no association between SBP and severe COVID-19 in untreated individuals, however, this is likely due to smaller numbers within the untreated individuals and hence a lack of power to detect any effects (S9 Table in S1 File). Sensitivity analyses also showed that compared to a ‘normal’ DBP (80-90mmHg), having a DBP higher than 90mmHg was associated with a higher odds of severe COVID-19 (S10 Table in S1 File).

**Fig 3:**
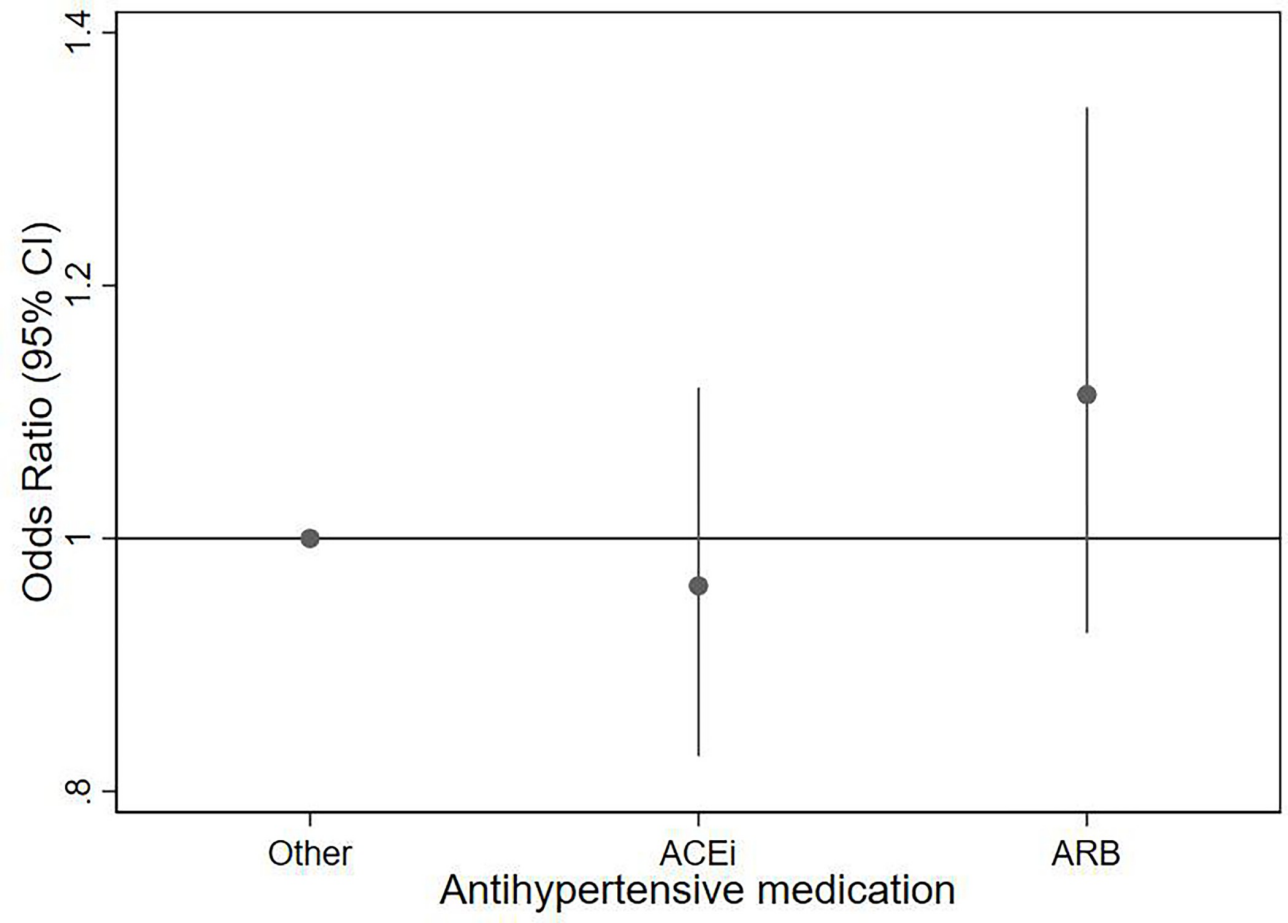
Odds ratio with 95% confidence interval for the fully adjusted model* for effect of type of antihypertensive medication^†^ on the risk of severe COVID-19 in individuals with hypertension who are treated with antihypertensive medications. *Model adjusted for Townsend deprivation index (measure of socioeconomic status (SES)), diabetes, smoking, ethnicity, age group, BMI category, CV comorbidity, stroke, male sex and SBP. ^†^Antihypertensive medications were categorised into ‘other’, ACEi and ARB, with the reference category defined as: ‘other’.

There did not appear to be any difference in the risk of severe COVID-19 between individuals taking ACEi and those taking ARBs, or other antihypertensive medications (Fig 3 and S11 Table in S1 File). This was irrespective of whether combination therapy or monotherapy was considered, or when considering only those individuals in whom SBP measurements were available from 2016 onwards (S12-S14 Tables in S1 File). Modelling time to severe COVID-19 showed comparable results (S15 Table in S1 File).

### Impact of broadening the classification of hypertension

The above analyses were repeated on a dataset of COVID-19 positive individuals, which additionally included any individual whose most recent BP measurement ≥ SBP 140mmHg or DBP 90mmHg, irrespective of their ICD10 coding status. Under this broader classification, 54.7% (n = 8,831) individuals had hypertension, of whom 53.8% (n = 4,7474) were treated. Hypertension was present in 70.9% (n = 2,540) of individuals with severe COVID-19 versus 50.1% (n = 6,291) of individuals without severe COVID-19 and having hypertension was associated with a 38% higher risk of severe COVID-19 (OR): 1.38 (1.26, 1.51)) (S16 Table in S1 File). After adjusting for potential intermediate variables, the direct effect of hypertension was similar to the total effect of hypertension (OR: 1.33 (1.22, 1.46)) (S17 Table in S1 File). The effects of SBP and antihypertensive medications on the risk of severe COVID-19 were comparable between the different definitions of hypertension used for these analyses (S17 Table in S1 File).

## Discussion

Our observational study on over 16,000 individuals who tested positive for COVID-19 from the UK Biobank showed that individuals with hypertension had over twice the risk of developing severe COVID-19 compared with non-hypertensive individuals. Although attenuated, the effect of hypertension remained after adjusting for confounding variables. In treated hypertensives, a SBP >150mmHg was associated with a higher risk of severe COVID-19 compared with the reference SBP level (120-129mmHg), as was a SBP<120mmHg. However, the type of antihypertensive medication did not appear to influence the risk of severe COVID-19.

The majority of the effect of hypertension on development of severe COVID-19 was direct. However, a modest proportion of the effect was mediated via CV comorbidities such as PVD, MI, CHD, arrhythmias and stroke. Broadening our classification of hypertension to additionally include those individuals with SBP ≥140mmHg or DBP ≥90mmHg showed that hypertension had a slightly higher association with severe COVID-19, compared with the association based on our original hypertension classification. Interestingly, very little of this effect was mediated via CV comorbidities, whereas age, smoking, being male, having a lower SES, higher BMI, being diabetic, as well as having hypertension were all associated with a higher risk of severe COVID-19.

Our study also suggests that there are further effects influencing the severity of COVID-19 beyond a dichotomous diagnosis of hypertension. Individuals with a higher than target SBP, may be less healthy, less active, suffering more severe hypertension or have developed drug resistant hypertension, all suggesting that the effects of hypertension have already had detrimental physiological effects on the CV system, which in turn may offer some explanation for the higher risk of severe COVID-19 with uncontrolled SBP. Our analyses also showed that the association between SBP and severe COVID-19 was J-shaped, with a SBP<120mmHg associated with a 36% higher risk of severe COVID-19 in treated hypertensive individuals. This may be due to reverse causality, where low SBP levels may indicate poorer health, such that the occurrence of severe COVID-19 may be related to underlying disease rather than the level of SBP *per se*. Indeed, J-Shaped associations between SBP and CV event rates and mortality have been demonstrated previously [24]. Nevertheless, the J-shaped association observed here remained after multiple adjustments, including presence of known CV comorbidities, suggesting a possible “real” effect of low SBP on severe COVID-19, at least in treated hypertensive individuals. Interestingly, this association did not exist in untreated individuals with hypertension, possibly due to fewer hypertensive individuals left untreated (and therefore an issue with power) or those untreated may have a more recent onset of hypertension (and therefore an issue with exposure).

A key rationale for undertaking our study was the premise that alterations in circulating levels of ACE2, which the SARS-COV2 virus binds to for cell entry, may alter susceptibility to severe COVID-19. Indeed, upregulation of ACE2 occurs in hypertensive individuals treated with ACEi or ARBs and expression of ACE2 is increased in diabetics treated with ACEi or ARBs (2), hence concerns over prescribing these drugs during the coronavirus pandemic. It should be noted, however, that the evidence concerning upregulation of ACE2 with ARBs, in particular, is inconsistent, and varies by organ and receptor blocker (27). We found no association between use of ACEi or ARBs and severe COVID-19, in concordance with published data showing little association between RAAS drugs and the risk of severe COVID-19 compared with other BP lowering medications or no medications [14, 20, 25–27]. Taken together, these observations suggest that circulating ACE2 levels may not necessarily affect the risk of severe COVID-19, at least in hypertensive individuals, although we did not assess circulating levels of ACE2 in our study population.

The strengths of this study include the large cohort with detailed demographic variables, as well as large numbers of primary health care records available to be linked to the database providing recent covariate, prescription and diagnostic data. To our knowledge, this is the first study to focus on the effects of SBP, in addition to the effects of hypertension, *per se*. Many of the previous studies assessing the effects of RAAS drugs and COVID-19 have compared groups taking RAAS drugs with those taking no medications. However, we compared the effects of RAAS drugs with other BP lowering medications, more accurately reflecting the hypertensive population.

We believe that censoring these data in early 2021 helped to reduce confounding from mutations that have increased transmissibility but reduced severity of disease, as well as effects of widespread vaccination and outpatient treatments. There are however, several limitations to the study and the data used for these analyses. The UK Biobank population is generally ‘healthier’ than the general UK population and has relatively few participants from ethnic minority groups, so generalisations to the wider UK population need to be undertaken with caution. Moreover, it was shown early on in the pandemic that the incidence of severe COVID-19 was 27% lower in the UK Biobank population compared with the wider population in England [28]. However, Batty et al. showed that despite risk factor levels and mortality rates being more favourable in UK Biobank, risk factor associations seem to be generalisable; it is unlikely that the association measures are biased due to any effect of this [29]. There is also some concern for selection bias in these analyses since COVID-19, the outcome of interest, drives the analysable population [30]. We have tried to minimise this by using the population who tested positive, as opposed to all tested for COVID-19. Indeed, the summary statistics and proportions of hypertension and co-morbidities were similar in the analysable population and the whole UK Biobank cohort, suggesting little concern for selection bias.

There are vast variations in testing frequency across the UK, so analysing the subset of those individuals who tested positive for COVID-19 would not eliminate all bias. Testing frequency and the incidence of COVID-19 also changed during the year of follow-up. However, we did not find any interaction with hypertension and testing season and our time-to-event analyses gave comparable results. Moreover, the findings of a previous study did not change substantially when severe COVID-19 patients were stratified by date of diagnosis [20]. Finally, we have taken positive COVID-19 cases from linked primary care data, however we could not identify severe cases from these, thus our results may somewhat underestimate the number of severe COVID-19 cases in our dataset. However, we assumed that the majority of severe cases were captured in the UK Biobank COVID-19 lab tests and from HES data.

Due to the nature of the SARS-Cov2 virus affecting individuals in vastly differing ways, with many asymptomatic infected individuals, we focused on individuals who were hospitalised with COVID-19 and are likely to have suffered from severe symptoms from COVID-19. There are, however, additional limitations to the interpretations of these results. There may be individuals who tested positive for COVID-19, not as an in-patient, but who later were admitted to hospital, or who tested negative but later tested positive and admitted to hospital. Additionally, all inpatients were tested for COVID-19, so in some cases the primary reason for being admitted to an inpatient unit may not have been COVID-19.

Even though the mortality rate due to COVID-19 has been hugely reduced over the last year due to mutation, vaccination and effective treatments, this study highlights the importance of hypertension as a risk factor for COVID-19, potentially increasing susceptibility to SARS-Cov2 via the RAAS. In particular, our data suggest that further research is needed into the mechanisms driving hypertension as a risk factor for COVID-19 in case of novel, more severe strains or other viruses in the future.

Hypertension is a risk factor for COVID-19, the association between hypertension and COVID-19 was amplified if the individuals were treated and their BP remained uncontrolled. The odds of severe COVID-19 was not affected by medication type.

## Supporting information

S1 Checklist STROBE statement—checklist of items that should be included in reports of observational studies. (DOCX)

S1 File (DOCX)
