# Supplementary material for: Primary hypertension, anti-hypertensive medications and the risk of severe COVID-19 in UK Biobank
Source: PLoS One. 2022 Nov 9;17(11):e0276781. doi: 10.1371/journal.pone.0276781 (PMC9645600; doi:10.1371/journal.pone.0276781)
Supplement: S1 Checklist — (DOCX) [file pone.0276781.s001.docx]

STROBE Statement—checklist of items that should be included in reports of observational studies

|  | Item No. | Recommendation | Page  No. | Relevant text from manuscript |
| --- | --- | --- | --- | --- |
| **Title and abstract** | 1 | (*a*) Indicate the study’s design with a commonly used term in the title or the abstract | 2 | Analyses were conducted using data from the UK Biobank and linked health records. |
|  |  | (*b*) Provide in the abstract an informative and balanced summary of what was done and what was found | 2 | Analyses were conducted using data from the UK Biobank and linked health records. Logistic regression models were fitted to assess the impact of hypertension, systolic blood pressure (SBP) and medications on the risk of severe COVID-19.  16,134 individuals tested positive for severe acute respiratory syndrome-coronavirus, 22% (n=3,584) developed severe COVID-19 and 40% (n=6,517) were hypertensive. Hypertension was associated with 22% higher odds of severe COVID-19 (Odds ratio (OR) 1.22; 95% confidence interval (CI) 1.12, 1.33), compared with normotension after adjusting for confounding variables. In those taking anti-hypertensive medications, elevated SBP showed a dose-response relationship with severe COVID-19 (150-159mmHg versus 120-129mmHg (OR 1.91; 95% CI 1.44, 2.53), >180+mmHg versus 120-129mmHg (OR 1.93; 95% CI 1.06, 3.51)). SBP <120mmHg was associated with greater odds of severe COVID-19 (OR 1.40; 95% CI 1.11, 1.78). Angiotensin-converting enzyme inhibitors or angiotensin-II receptor blockers were not associated with altered risk of severe COVID-19. |
| Introduction | | | |  |
| Background/rationale | 2 | Explain the scientific background and rationale for the investigation being reported | 3 | Indeed, a meta-analysis and systematic review exploring COVID-19 and associated comorbidities, found that hypertension was more prevalent in severe and fatal cases (48%) compared with all cases of COVID-19 (25%) [9]. However, previous studies have not adjusted consistently for key confounding factors such as age, ethnicity and socioeconomic status, which are themselves predictors of hypertension and severe COVID-19 [1,7,9]. It also remains unclear whether, amongst hypertensive individuals, the risk of COVID-19 varies according to the level of systolic blood pressure (SBP) and/or type of antihypertensive medication. Many studies have now examined associations between Angiotensin-converting enzyme inhibitors (ACEi) or angiotensin-II receptor blockers (ARBs) and the risk of COVID-19, but not with a specific focus on hypertensive individuals. |
| Objectives | 3 | State specific objectives, including any prespecified hypotheses | 4 | The aim of this study was to estimate the risk of severe COVID-19 between individuals with and without hypertension and explore whether any increased risk of severe COVID-19 was dependent on the absolute BP and/or type of antihypertensive treatment. |
| Methods | | | |  |
| Study design | 4 | Present key elements of study design early in the paper | 4 | Participants from the UK Biobank, a large population-based cohort study, with linked primary health care records, death records and COVID-19 test results were included in these analyses. |
| Setting | 5 | Describe the setting, locations, and relevant dates, including periods of recruitment, exposure, follow-up, and data collection | 4 & 6 | We used 04/04/2021 as the censor date for all records and test results used for these analyses.  The UK Biobank is a longitudinal prospective study, initiated in 2006 and has recruited over 500,000 men and women aged between 40 and 69 years from the UK. It was established to investigate diseases in middle and older ages. At baseline (2006-2010) and over two further follow-up visits, a vast array of data, including physical measures, lifestyle data and biological samples, were collected.    From March 16^th^ 2020, COVID-19 test results from SARS-COV2 polymerase chain reaction (PCR)-based swab tests, provided by Public Health England (PHE), were available to be linked to UK Biobank data. |
| Participants | 6 | (*a*) *Cohort study*—Give the eligibility criteria, and the sources and methods of selection of participants. Describe methods of follow-up  *Case-control study*—Give the eligibility criteria, and the sources and methods of case ascertainment and control selection. Give the rationale for the choice of cases and controls  *Cross-sectional study*—Give the eligibility criteria, and the sources and methods of selection of participants | 6 | Individuals known to have been lost to follow up or who died prior to 16 March 2020 (initiation date of COVID-19 testing in UK Biobank) were excluded from the analyses.  To minimise any misclassification bias and bias due to shielding, analyses were performed only on individuals who had a confirmed positive test result for or were diagnosed with COVID-19, making the assumption that all included participants had been exposed to the SARS-COV2 virus. |
|  |  | (*b*) *Cohort study*—For matched studies, give matching criteria and number of exposed and unexposed  *Case-control study*—For matched studies, give matching criteria and the number of controls per case |  |  |
| Variables | 7 | Clearly define all outcomes, exposures, predictors, potential confounders, and effect modifiers. Give diagnostic criteria, if applicable | 5-6 | For the main analyses, hypertension status was determined using primary and secondary ICD10 codes for essential (primary) hypertension (I10) obtained from the UK Biobank cohort data including self-reported data, hospital episodes from Hospital Episode Statistics (HES) data as well as primary health care records for the individuals, where available.  An individual was defined as taking BP lowering medication if they were prescribed BP lowering medications on their most recent prescription record. SBP, diastolic BP (DBP) and body mass index (BMI) were obtained from the primary care clinical records (see data supplement for code lists), where available, otherwise from the UK Biobank database, taking the BP reading closest to the analysis baseline (16/03/2020). Current smokers were those with a record of being a current smoker at their most recent UK Biobank visit. The Townsend deprivation index (TDI) calculated immediately prior to the UK Biobank baseline visit was used as a measure of socio-economic status (SES). Ethnicity was dichotomised into white and non-white (due to small numbers) and sex was taken from the baseline UK Biobank visit. All data were assumed to be true at the analysis baseline. Comorbidity was defined as any of the following: peripheral vascular disease (PVD), myocardial infarction (MI), coronary heart disease (CHD) (excluding hypertension), angina, heart failure (HF) and arrhythmia or stroke at any one of the UK Biobank follow-up visits, prevalent at baseline or history of a CV comorbidity based on International Classification of Diseases 10 (ICD10) codes from hospital inpatient episodes or from primary health care records using primary or secondary positions of the diagnostic codes.  Death due to COVID-19 was obtained using ICD10 codes from the death records, primary care records and hospital inpatient records. |
| Data sources/ measurement | 8* | For each variable of interest, give sources of data and details of methods of assessment (measurement). Describe comparability of assessment methods if there is more than one group | *4* | At baseline (2006-2010) and over two further follow-up visits, a vast array of data, including physical measures, lifestyle data and biological samples, were collected. The UK Biobank additionally released primary care records for approximately 409,000 individuals for use solely in COVID-19 research, providing a wealth of further and longer-term health outcomes. |
| Bias | 9 | Describe any efforts to address potential sources of bias | 6 | To minimise any misclassification bias and bias due to shielding, analyses were performed only on individuals who had a confirmed positive test result for or were diagnosed with COVID-19, making the assumption that all included participants had been exposed to the SARS-COV2 virus. |
| Study size | 10 | Explain how the study size was arrived at | 8 | 16,134 individuals who tested positive for, who were diagnosed with or who died due to COVID-19, with complete covariate data, were included in the analyses. |

Continued on next page

| Quantitative variables | 11 | Explain how quantitative variables were handled in the analyses. If applicable, describe which groupings were chosen and why | 7 | The linearity of continuous variables was assessed and all two-way interactions were considered and included only if the coefficient of the independent variable was altered significantly. Since the relationship between SBP and severe COVID-19 was non-linear, SBP and other continuous variables were categorised for ease of interpretation by the reader. Age was categorised in to decades: <60, ≥60 to <70, ≥70 to <80 and ≥80 years. BMI was categorised based on general BMI classifications: <25, ≥25 to <30, ≥30 to <35 and ≥35 kg/m^2^, classified as normal, overweight, class 1 obesity and class 2 obesity, respectively. SBP was categorised by 10mmHg ranges, starting from <120mmHg up to 180+mmHg, with the reference category defined as: 120-129mmHg, based on data from the SPRINT study demonstrating that intensive SBP lowering to below 120mmHg, as compared with the traditional threshold of 140mmHg, is beneficial [23]. DBP was categorised by 10mmHg ranges, starting from <60mmHg up to 100+mmHg with 80-90mmHg being the reference category. CRP was categorised into four categories: <3 mg/L, ≥3 to <10 mg/L, ≥10 to <100 mg/L and 100+ mg/L, classified as normal, minor elevations, moderately elevated and elevated. |
| --- | --- | --- | --- | --- |
| Statistical methods | 12 | (*a*) Describe all statistical methods, including those used to control for confounding | 7 | Logistic regression was used to assess the association between hypertension, SBP or antihypertensive medication and the odds of severe COVID-19. All analyses were adjusted for age, sex, BMI, ethnicity, smoking status, diabetes status, SES and inflammation (c-reactive protein (CRP)) as these were proposed as potential confounders. To assess the direct effect of hypertension on COVID-19, we adjusted for intermediate variables on the causal pathway between hypertension and severe COVID-19, these included CV comorbidities and Stroke. These variables were additionally adjusted for when modelling SBP and type of antihypertensive medications. |
|  |  | (*b*) Describe any methods used to examine subgroups and interactions | 7 | all two-way interactions were considered and included only if the coefficient of the independent variable was altered significantly. |
|  |  | (*c*) Explain how missing data were addressed | 7 | Analyses were performed on the subset of individuals with complete cases for the required variables. |
|  |  | (*d*) *Cohort study*—If applicable, explain how loss to follow-up was addressed  *Case-control study*—If applicable, explain how matching of cases and controls was addressed  *Cross-sectional study*—If applicable, describe analytical methods taking account of sampling strategy |  | NA |
|  |  | (*e*) Describe any sensitivity analyses | 8 | we repeated the key analyses using a cox proportional hazard model, with time to severe COVID-19 as the outcome. This allowed for variations in the underlying rate of COVID-19 infection over time. A number of sensitivity analyses were then considered including: (i) broadening our classification of hypertension to additionally include any individual whose most recent BP measurement indicated SBP ≥140mmHg or DBP ≥90mmHg (ii) assessing the association between hypertension with death due to COVID-19 as the outcome, (iii) considering the effect of SBP on the odds of severe COVID-19 in both treated and untreated individuals with hypertension, (iv) using the average SBP since 2016, (v) considering treatment by mono- or combination therapy, as well as considering individuals who had not switched between ACEi and ARB treatment. We also looked at the association between DBP and severe COVID-19. |
| Results | | | | |
| Participants | 13* | (a) Report numbers of individuals at each stage of study—eg numbers potentially eligible, examined for eligibility, confirmed eligible, included in the study, completing follow-up, and analysed | Figure 1 |  |
|  |  | (b) Give reasons for non-participation at each stage | Figure 1 |  |
|  |  | (c) Consider use of a flow diagram | Figure 1 | See Figure 1 in manuscript. |
| Descriptive data | 14* | (a) Give characteristics of study participants (eg demographic, clinical, social) and information on exposures and potential confounders | 10, table 1 & table 2 | The mean (SD) age was 65.3 (8.7) years, 47% were male, 90% were white and 40% were diagnosed with essential hypertension at the analysis baseline. Of the 16,134 individuals included in the analyses, SBP values were obtained from primary care records for 14,253 (88%) individuals. Severe COVID-19 occurred in 3,584 (22%) individuals, of whom 1,061 (30%) died. Individuals with severe COVID-19 were marginally older, more likely to be male (57%) and more deprived. They were also more likely to be hypertensive (56%, n=2,024) compared with individuals without severe COVID-19 (36%, n=4,493) and a greater proportion of individuals with severe COVID-19 had CV comorbidities. A total of 6,517 (40%) individuals had a diagnosis of essential hypertension, of whom 4,391 (67%) were treated (41% monotherapy (n=1,798), 59% combination therapy (n=2,593)) and 2,126 (33%) were untreated.  See Table 1 and table 2. |
|  |  | (b) Indicate number of participants with missing data for each variable of interest | NA | NA – The complete case population was used. |
|  |  | (c) *Cohort study*—Summarise follow-up time (eg, average and total amount) | 4-5 & 12 | Analysis baseline: (16/03/2020).  We used 04/04/2021 as the censor date for all records and test results used for these analyses.  over a 384 day follow-up period |
| Outcome data | 15* | *Cohort study*—Report numbers of outcome events or summary measures over time | *8* | Severe COVID-19 occurred in 3,584 (22%) individuals, of whom 1,061 (30%) died. |
|  |  | *Case-control study—*Report numbers in each exposure category, or summary measures of exposure |  |  |
|  |  | *Cross-sectional study—*Report numbers of outcome events or summary measures |  |  |
| Main results | 16 | (*a*) Give unadjusted estimates and, if applicable, confounder-adjusted estimates and their precision (eg, 95% confidence interval). Make clear which confounders were adjusted for and why they were included | 11-12 | The unadjusted odds ratio (OR) of the association between hypertension and severe COVID-19 was 2.33 (95% CI: 2.16, 2.51). Adjusting for age greatly attenuated the association (age adjusted OR: 1.47 (1.35, 1.59)), as did diabetes (OR: 2.00 (1.85, 2.16)) and BMI (OR: 2.20 (2.04, 2.38)) (Table S1). After concurrently adjusting for all potential confounders, the adjusted OR was 1.22 (1.12, 1.33) (p<0.01).  In hypertensive individuals receiving antihypertensive medications, there was a J-shaped relationship between the level of BP and risk of severe COVID-19, using a SBP level of 120-129 mmHg as a reference (Figure 2). A SBP of 150-159mmHg was associated with a 91% higher risk of suffering severe COVID-19 compared with a SBP of 120-129mmHg (OR: 1.91 (1.44, 2.53), p<0.001).  There did not appear to be any difference in the risk of severe COVID-19 between individuals taking ACEi and those taking ARBs. |
|  |  | (*b*) Report category boundaries when continuous variables were categorized |  | See item number 11. |
|  |  | (*c*) If relevant, consider translating estimates of relative risk into absolute risk for a meaningful time period |  | NA |

Continued on next page

| Other analyses | 17 | Report other analyses done—eg analyses of subgroups and interactions, and sensitivity analyses | 12-14 | A sensitivity analysis revealed that the effect of hypertension on the hard outcome of death due to COVID-19 was similar to development of severe COVID-19 (OR: 1.17 (1.01, 1.36); Table S1). After further adjusting for possible effect mediators (CV comorbidities and stroke), there was a modest attenuation in the association between hypertension and severe COVID-19 (OR: 1.15 (1.05, 1.26); Table S2).  These results were similar when time to severe COVID-19 was analysed using a time-to-event model, over a 384 day follow-up period.  The results looking at the effect of SBP of the odds of severe COVID-19 were similar when restricted to individuals treated by combination therapy, but were less apparent when restricted to individuals taking monotherapy only and when averaging the SBP since 2016 (Tables S6, S7 & S8, respectively). Analyses including all hypertensive individuals further stratified by whether or not they were taking antihypertensive medications showed that there was no association between SBP and severe COVID-19 in untreated individuals. Sensitivity analyses also showed that compared to a ‘normal’ DBP (80-90mmHg), having a DBP higher than 90mmHg was associated with a higher odds of severe COVID-19.  There did not appear to be any difference in the risk of severe COVID-19 between individuals taking ACEi and those taking ARBs, or other antihypertensive medications (Figure 3, Table S11). This was irrespective of whether combination therapy or monotherapy was considered, or when considering only those individuals in whom SBP measurements were available from 2016 onwards.  Broadening the definition of hypertension to additionally include any individual whose most recent BP measurement ≥ SBP 140mmHg or DBP 90mmHg, irrespective of their ICD10 coding status showed that having hypertension was associated with a 38% higher risk of severe COVID-19 (OR): 1.38 (1.26, 1.51)). After adjusting for potential intermediate variables, the direct effect of hypertension was similar to the total effect of hypertension (OR: 1.33 (1.22, 1.46)) (Table S17). The effects of SBP and antihypertensive medications on the risk of severe COVID-19 were comparable between the different definitions of hypertension used for these analyses. |
| --- | --- | --- | --- | --- |
| Discussion | | | | |
| Key results | 18 | Summarise key results with reference to study objectives | 14 | Our observational study on over 17,000 individuals who tested positive for COVID-19 from the UK Biobank showed that individuals with hypertension had over twice the risk of developing severe COVID-19 compared with non-hypertensive individuals. Although attenuated, the effect of hypertension remained after adjusting for confounding variables. In treated hypertensives, a SBP >150mmHg was associated with a higher risk of severe COVID-19 compared with the reference SBP level (120-129mmHg), as was a SBP<120mmHg. However, the type of antihypertensive medication did not appear to influence the risk of severe COVID-19. |
| Limitations | 19 | Discuss limitations of the study, taking into account sources of potential bias or imprecision. Discuss both direction and magnitude of any potential bias | 16-17 | The UK Biobank population is generally ‘healthier’ than the general UK population and has relatively few participants from ethnic minority groups, so generalisations to the wider UK population need to be undertaken with caution. Moreover, it was shown early on in the pandemic that the incidence of severe COVID-19 was 27% lower in the UK Biobank population compared with the wider population in England [28]. However, Batty et al. showed that despite risk factor levels and mortality rates being more favourable in UK Biobank, risk factor associations seem to be generalisable; it is unlikely that the association measures are biased due to any effect of this [29]. There is also some concern for selection bias in these analyses since COVID-19, the outcome of interest, drives the analysable population [30]. We have tried to minimise this by using the population who tested positive, as opposed to all tested for COVID-19. Indeed, the summary statistics and proportions of hypertension and co-morbidities were similar in the analysable population and the whole UK Biobank cohort, suggesting little concern for selection bias.  There are vast variations in testing frequency across the UK, so analysing the subset of those individuals who tested positive for COVID-19 would not eliminate all bias. Testing frequency and the incidence of COVID-19 also changed during the year of follow-up. However, we did not find any interaction with hypertension and testing season and our time-to-event analyses gave comparable results. Moreover, the findings of a previous study did not change substantially when severe COVID-19 patients were stratified by date of diagnosis [20]. Finally, we have taken positive COVID-19 cases from linked primary care data, however we could not identify severe cases from these, thus our results may somewhat underestimate the number of severe COVID-19 cases in our dataset. However, we assumed that the majority of severe cases were captured in the UK Biobank COVID-19 lab tests and from HES data.  Due to the nature of the SARS-Cov2 virus affecting individuals in vastly differing ways, with many asymptomatic infected individuals, we focused on individuals who were hospitalised with COVID-19 and are likely to have suffered from severe symptoms from COVID-19. There are, however, additional limitations to the interpretations of these results. There may be individuals who tested positive for COVID-19, not as an in-patient, but who later were admitted to hospital, or who tested negative but later tested positive and admitted to hospital. Additionally, all inpatients were tested for COVID-19, so in some cases the primary reason for being admitted to an inpatient unit may not have been COVID-19. |
| Interpretation | 20 | Give a cautious overall interpretation of results considering objectives, limitations, multiplicity of analyses, results from similar studies, and other relevant evidence | 18 | Even though the mortality rate due to COVID-19 has been hugely reduced over the last year due to mutation, vaccination and effective treatments, this study highlights the importance of hypertension as a risk factor for COVID-19, potentially increasing susceptibility to SARS-Cov2 via the RAAS. In particular, our data suggest that further research is needed into the mechanisms driving hypertension as a risk factor for COVID-19 in case of novel, more severe strains or other viruses in the future.  Hypertension is a risk factor for COVID-19, the association between hypertension and COVID-19 was amplified if the individuals were treated and their BP remained uncontrolled. The odds of severe COVID-19 was not affected by medication type. |
| Generalisability | 21 | Discuss the generalisability (external validity) of the study results | 16-17 | The UK Biobank population is generally ‘healthier’ than the general UK population and has relatively few participants from ethnic minority groups, so generalisations to the wider UK population need to be undertaken with caution. |
| Other information | |  | | |
| Funding | 22 | Give the source of funding and the role of the funders for the present study and, if applicable, for the original study on which the present article is based |  | HP is supported by a Cambridge BHF CRE non-clinical PhD studentship. AW is part ofthe BigData@Heart Consortium, funded by the Innovative Medicines Initiative-2 Joint Undertaking under grant agreement No 116074. AW is supported by the BHF-Turing Cardiovascular Data Science Award (BCDSA\100005) and by core funding from UK MRC (MR/L003120/1) and BHF (RG/13/13/30194; RG/18/13/33946). CMM, IBW and AW acknowledge funding support from the NIHR Cambridge Biomedical Research Centre (BRC-1215-20014). The views expressed are those of the authors and not necessarily those of the NIHR or the Dept Health and Social Care. PSS is supported by the Biomedical Research Centre Award to Imperial College Healthcare NHS Trust. YBS is partially funded by the NIHR Applied Research Collaboration (ARC) West |

*Give information separately for cases and controls in case-control studies and, if applicable, for exposed and unexposed groups in cohort and cross-sectional studies.

**Note:** An Explanation and Elaboration article discusses each checklist item and gives methodological background and published examples of transparent reporting. The STROBE checklist is best used in conjunction with this article (freely available on the Web sites of PLoS Medicine at http://www.plosmedicine.org/, Annals of Internal Medicine at http://www.annals.org/, and Epidemiology at http://www.epidem.com/). Information on the STROBE Initiative is available at www.strobe-statement.org.
