# Supplementary material for: Primary hypertension, anti-hypertensive medications and the risk of severe COVID-19 in UK Biobank
Source: PLoS One. 2022 Nov 9;17(11):e0276781. doi: 10.1371/journal.pone.0276781 (PMC9645600; doi:10.1371/journal.pone.0276781)
Supplement: S1 File — (DOCX) [file pone.0276781.s002.docx]

**DATA SUPPLEMENT**

| **Table S1. Odds ratio (OR) and 95% confidence interval (CI) for the total effect of essential (primary) hypertension and potential confounders on the odds of severe COVID-19 and death due to COVID-19 (N=16,134)** | | | | | | | | | |
| --- | --- | --- | --- | --- | --- | --- | --- | --- | --- |
|  | **Outcome: Severe COVID-19** | | | **Outcome: Severe COVID-19** | | | **Outcome: COVID-19 death** | | |
| **Covariate** | **OR*** | **95% CI** | **p-value** | **OR^†^** | **95% CI** | **p-value** | **OR^*^** | **95% CI** | **p-value** |
| **Hypertension** | 1.22 | (1.12, 1.33) | <0.001 | 2.33 | (2.16, 2.51) | <0.001 | 1.17 | (1.01, 1.36) | 0.036 |
| **Ref: SES (Least Deprived)** | 1 | - | - | 2.30 | (2.13, 2.48) | <0.001 | 1 | - | - |
| **SES (Less Deprived)** | 1.03 | (0.91, 1.17) | 0.635 |  |  |  | 1.21 | (0.97, 1.50) | 0.089 |
| **SES (More Deprived)** | 1.14 | (1.01, 1.29) | 0.030 |  |  |  | 1.26 | (1.02, 1.56) | 0.033 |
| **SES (Most Deprived)** | 1.21 | (1.08, 1.36) | 0.002 |  |  |  | 1.55 | (1.27, 1.89) | <0.001 |
| **Sex (Male)** | 1.47 | (1.35, 1.59) | <0.001 | 2.25 | (2.08, 2.42) | <0.001 | 1.80 | (1.57, 2.07) | <0.001 |
| **Ref: BMI (<25)** | 1 | - | - | 2.20 | (2.04, 2.38) | <0.001 | 1 | - | - |
| **BMI (25-30)** | 1.12 | (1.01, 1.24) | 0.029 |  |  |  | 0.97 | (0.82, 1.15) | 0.722 |
| **BMI (30-35)** | 1.27 | (1.12, 1.343) | <0.001 |  |  |  | 1.12 | (0.92, 1.37) | 0.256 |
| **BMI (35+)** | 1.83 | (1.58, 2.12) | <0.001 |  |  |  | 1.80 | (1.42, 2.26) | <0.001 |
| **Diabetes** | 1.60 | (1.44, 1.78) | <0.001 | 2.00 | (1.85, 2.16) | <0.001 | 1.61 | (1.37, 1.88) | <0.001 |
| **Smoker** | 1.50 | (1.33, 1.70) | <0.001 | 2.34 | (2.17, 2.52) | <0.001 | 1.52 | (1.25, 1.84) | <0.001 |
| **Ethnicity (Non-white)** | 0.98 | (0.84, 1.13) | 0.741 | 2.35 | (2.18, 2.53) | <0.001 | 1.10 | (0.85, 1.40) | 0.468 |
| **Ref: Age (<60)** | 1 | - | - | 1.47 | (1.35, 1.59) | <0.001 | 1 | - | - |
| **Age (60-70)** | 1.63 | (1.44, 1.83) | <0.001 |  |  |  | 4.39 | (3.11, 6.35) | <0.001 |
| **Age (70-80)** | 4.84 | (4.32, 5.43) | <0.001 |  |  |  | 19.32 | (14.00, 27.48) | <0.001 |
| **Age (80+)** | 9.55 | (7.96, 11.46) | <0.001 |  |  |  | 41.53 | (28.90, 61.12) | <0.001 |
| **Ref: CRP (<3 mg/L)** | 1 |  |  | 2.29 | (2.12, 2.47) | <0.001 | 1 |  |  |
| **CRP (3-10 mg/L)** | 1.19 | (1.08, 1.31) | <0.001 |  |  |  | 1.22 | (1.05, 1.42) | 0.009 |
| **CRP (10-100 mg/L)** | 1.58 | (1.35, 1.84) | <0.001 |  |  |  | 1.67 | (1.33, 2.09) | <0.001 |
| **CRP (100+ mg/L)** | 2.89 | (1.27, 6.49) | 0.010 |  |  |  | 1.87 | (0.51, 5.40) | 0.287 |
| *OR adjusted for all covariates listed in the table  ^†^OR for hypertension adjusted for each covariate in turn | | | |  |  |  |  |  |  |

| **Table S2. Odds ratios (OR) and 95% confidence interval (CI) for the total effect of essential (primary) hypertension and potential confounders and mediators (CV Comorbidities^*^ and stroke) on the odds of severe COVID-19 (N=16,134)** | | | |
| --- | --- | --- | --- |
| **Covariate** | **OR^†^** | **95% CI** | **p-value** |
| **Hypertension** | 1.15 | (1.05, 1.26) | 0.003 |
| **Ref: SES (Least Deprived)** | 1 | - | - |
| **SES (Less Deprived)** | 1.02 | (0.90, 1.16) | 0.704 |
| **SES (More Deprived)** | 1.13 | (1.00, 1.28) | 0.044 |
| **SES (Most Deprived)** | 1.19 | (1.06, 1.34) | 0.004 |
| **Sex (Male)** | 1.43 | (1.31, 1.55) | <0.001 |
| **Ref: BMI (<25)** |  |  |  |
| **BMI (25-30)** | 1.13 | (1.02, 1.25) | 0.022 |
| **BMI (30-35)** | 1.28 | (1.13, 1.44) | <0.001 |
| **BMI (35+)** | 1.84 | (1.59, 2.13) | <0.001 |
| **Diabetes** | 1.55 | (1.39, 1.72) | <0.001 |
| **Smoker** | 1.48 | (1.31, 1.67) | <0.001 |
| **Ethnicity (Non-White)** | 0.99 | (0.85, 1.14) | 0.880 |
| **Ref: Age (<60)** | 1 | - | - |
| **Age (60-70)** | 1.60 | (1.42, 1.80) | <0.001 |
| **Age (70-80)** | 4.55 | (4.05, 5.12) | <0.001 |
| **Age (80+)** | 8.69 | (7.23, 10.46) | <0.001 |
| **Ref: CRP (<3 mg/L)** | 1 | - | - |
| **CRP (3-10 mg/L)** | 1.18 | (1.07, 1.30) | <0.001 |
| **CRP (10-100 mg/L)** | 1.54 | (1.32, 1.79) | <0.001 |
| **CRP (100+ mg/L)** | 2.82 | (1.23, 6.33) | 0.012 |
| **CV Comorbidity^*^** | 1.30 | (1.18, 1.43) | <0.001 |
| **Stroke** | 1.47 | (1.18, 1.83) | 0.001 |
| *Not including stroke.  ^†^OR adjusted for all covariates in the table. | | | |

| **Table S3. Hazard ratio (HR) and 95% confidence interval (CI) for the total effect of essential (primary) hypertension and potential confounders on the odds of severe COVID-19 (N=16,134)** | | | |
| --- | --- | --- | --- |
| **Covariate** | **HR*** | **95% CI** | **p-value** |
| **Hypertension** | 1.17 | (1.09, 1.26) | <0.001 |
| **Ref: SES (Least Deprived)** | 1 | - | - |
| **SES (Less Deprived)** | 1.03 | (0.93, 1.15) | 0.531 |
| **SES (More Deprived)** | 1.14 | (1.03, 1.26) | 0.010 |
| **SES (Most Deprived)** | 1.20 | (1.09, 1.32) | <0.001 |
| **Ref: Age (<60)** | 1 | - | - |
| **Age (60-70)** | 1.59 | (1.42, 1.78) | <0.001 |
| **Age (70-80)** | 4.01 | (3.62, 4.44) | <0.001 |
| **Age (80+)** | 6.62 | (5.76, 7.61) | <0.001 |
| **Smoker** | 1.40 | (1.27, 1.54) | <0.001 |
| **Ref: BMI (<25)** | 1 | - | - |
| **BMI (25-30)** | 1.10 | (1.01, 1.20) | 0.026 |
| **BMI (30-35)** | 1.22 | (1.10, 1.35) | <0.001 |
| **BMI (35+)** | 1.65 | (1.46, 1.85) | <0.001 |
| **Diabetes** | 1.41 | (1.30, 1.53) | <0.001 |
| **Ethnicity (Non-white)** | 0.99 | (0.88, 1.12) | 0.872 |
| **Sex (Male)** | 1.40 | (1.31, 1.50) | <0.001 |
| **Ref: CRP (<3 mg/L)** | 1 |  |  |
| **CRP (3-10 mg/L)** | 1.14 | (1.06, 1.23) | 0.001 |
| **CRP (10-100 mg/L)** | 1.43 | (1.27, 1.61) | <0.001 |
| **CRP (100+ mg/L)** | 1.94 | (1.12, 3.34) | 0.018 |
| *HR adjusted for all covariates in the table. | | | |

| **Table S4. Odds ratio (OR) and 95% confidence interval (CI) of SBP and potential confounders on the odds of severe COVID-19 amongst individuals with hypertension who are treated with antihypertensive medications (N=4,391)** | | | |
| --- | --- | --- | --- |
| **Covariate** | **OR*** | **95% CI** | **p-value** |
| **SBP (<120mmHg)** | 1.40 | (1.11, 1.78) | 0.005 |
| **Ref: SBP (120-129mmHg)** | 1 | - | - |
| **SBP (130-139mmHg)** | 1.03 | (0.85, 1.24) | 0.756 |
| **SBP (140-149mmHg)** | 1.18 | (0.96, 1.46) | 0.117 |
| **SBP (150-159mmHg)** | 1.91 | (1.44, 2.53) | <0.001 |
| **SBP (160-169mmHg)** | 1.77 | (1.21, 2.58) | 0.003 |
| **SBP (170-179mmHg)** | 1.90 | (1.09, 3.31) | 0.023 |
| **SBP (180+mmHg)** | 1.93 | (1.06, 3.51) | 0.030 |
| **Ref: SES (Least Deprived)** | 1 | - | - |
| **SES (Less Deprived)** | 1.13 | (0.91, 1.40) | 0.281 |
| **SES (More Deprived)** | 1.25 | (1.02, 1.55) | 0.036 |
| **SES (Most Deprived)** | 1.33 | (1.09, 1.63) | 0.005 |
| **Diabetes** | 1.41 | (1.22, 1.63) | <0.001 |
| **Smoker** | 1.66 | (1.34, 2.05) | <0.001 |
| **Ethnicity (Non-White)** | 1.09 | (0.87, 1.36) | 0.443 |
| **Ref: Age (<60)** | 1 | - | - |
| **Age (60-70)** | 1.74 | (1.34, 2.26) | <0.001 |
| **Age (70-80)** | 4.18 | (3.27, 5.38) | <0.001 |
| **Age (80+)** | 7.54 | (5.48, 10.43) | <0.001 |
| **Ref: BMI (<25)** | 1 | - | - |
| **BMI (25-30)** | 1.08 | (0.89, 1.32) | 0.418 |
| **BMI (30-35)** | 1.13 | (0.92, 1.40) | 0.241 |
| **BMI (35+)** | 1.77 | (1.40, 2.25) | <0.001 |
| **CV Comorbidity^†^** | 1.27 | (1.11, 1.46) | 0.001 |
| **Stroke** | 1.53 | (1.16, 2.02) | 0.003 |
| **Sex (Male)** | 1.49 | (1.29, 1.71) | <0.001 |
| **Ref: CRP (<3 mg/L)** | 1 |  |  |
| **CRP (3-10 mg/L)** | 1.09 | (0.94, 1.27) | 0.260 |
| **CRP (10-100 mg/L)** | 1.45 | (1.14, 1.84) | 0.002 |
| **CRP (100+ mg/L)** | 2.17 | (0.5, 10.03) | 0.300 |
| *OR adjusted for all covariates in the table  ^†^Not including stroke  The number of individuals in each category of SBP differed somewhat, however the proportion of severe COVID-19 events in each category was relatively similar: SBP<120 (n=537 (12%), events=217 (40%)), SBP:120-129 (n=933 (21%), events=287 (31%)), SBP:130-139 (n=1,483 (34%), events=459 (31%)), SBP:140-149 (n=869 (20%), events=296 (34%)), SBP:150-159 (n=307 (7%), events=135 (44%)), SBP:160-169 (n=149 (3%), 65 (44%)), SBP:170-179 (n=61 (1%), events=29 (48%)) and SBP:180+ (n=52 (%), events = 24 (46%)). | | | |

| **Table S5. Odds ratio (OR) and 95% confidence interval (CI) of continuous SBP and potential confounders on the odds of severe COVID-19 amongst individuals with hypertension who are treated with antihypertensive medications (N=4,391)** | | | |
| --- | --- | --- | --- |
| **Covariate** | **OR*** | **95% CI** | **p-value** |
| **SBP** | 1.05 | (0.98, 1.13) | 0.186 |
| **SBP^2^** | 1.06 | (1.02, 1.10) | 0.001 |
| **Sex (Male)** | 1.45 | (1.27, 1.66) | <0.001 |
| **SES** | 1.04 | (1.02, 1.06) | <0.001 |
| **BMI** | 1.20 | (1.13, 1.27) | <0.001 |
| **Diabetes** | 1.40 | (1.21, 1.62) | <0.001 |
| **Current Smoker** | 1.69 | (1.37, 2.09) | <0.001 |
| **Ethnicity (Non-white)** | 1.13 | (0.90, 1.41) | 0.305 |
| **Age** | 1.09 | (1.08, 1.10) | <0.001 |
| **CV Comorbidity^†^** | 1.23 | (1.06, 1.41) | 0.005 |
| **Stroke** | 1.49 | (1.13, 1.97) | 0.005 |
| *OR adjusted for all covariates in the table  ^†^Not including stroke | | | |

| **Table S6. Odds ratio (OR) and 95% confidence interval (CI) of SBP and potential confounders on the odds of severe COVID-19 amongst individuals with hypertension who are treated with combination therapy (N=2,564)** | | | |
| --- | --- | --- | --- |
|  | **OR*** | **95% CI** | **p-value** |
| **SBP (<120mmHg)** | 1.54 | (1.14, 2.08) | 0.005 |
| **Ref: SBP (120-130mmHg)** | 1 |  |  |
| **SBP (130-140mmHg)** | 0.94 | (0.74, 1.2) | 0.629 |
| **SBP (140-150mmHg)** | 1.18 | (0.9, 1.55) | 0.219 |
| **SBP (150-160mmHg)** | 1.81 | (1.25, 2.62) | 0.002 |
| **SBP (160-170mmHg)** | 1.87 | (1.13, 3.08) | 0.015 |
| **SBP (170-180mmHg)** | 1.87 | (0.87, 3.98) | 0.105 |
| **SBP (180+mmHg)** | 2.25 | (1.04, 4.91) | 0.040 |
| **Ref: SES (Least Deprived)** | 1 |  |  |
| **SES (Less Deprived)** | 1.26 | (0.95, 1.68) | 0.11 |
| **SES (More Deprived)** | 1.38 | (1.05, 1.82) | 0.023 |
| **SES (Most Deprived)** | 1.54 | (1.19, 2) | 0.001 |
| **Sex (Male)** | 1.63 | (1.35, 1.96) | <0.001 |
| **Ref: BMI (<25)** | 1 |  |  |
| **BMI (25-30)** | 1.11 | (0.86, 1.43) | 0.434 |
| **BMI (30-35)** | 1.13 | (0.86, 1.49) | 0.393 |
| **BMI (35+)** | 1.93 | (1.41, 2.64) | <0.001 |
| **CV Comorbidity^†^** | 1.33 | (1.11, 1.6) | 0.002 |
| **Stroke** | 1.21 | (0.85, 1.71) | 0.295 |
| **Diabetes** | 1.42 | (1.18, 1.71) | <0.001 |
| **Current Smoker** | 1.71 | (1.3, 2.25) | <0.001 |
| **Ethnicity (Non-white)** | 1.11 | (0.82, 1.51) | 0.491 |
| **Ref: Age (<60)** | 1 |  |  |
| **Age (60-70)** | 1.93 | (1.35, 2.82) | <0.001 |
| **Age (70-80)** | 4.55 | (3.23, 6.53) | <0.001 |
| **Age (80+)** | 7.38 | (4.81, 11.48) | <0.001 |
| **Ref: CRP (<3mg/L)** |  |  |  |
| **CRP (3-10mg/L)** | 1.08 | (0.89, 1.31) | 0.439 |
| **CRP (10-100mg/L)** | 1.79 | (1.31, 2.46) | <0.001 |
| **CRP (100+ mg/L)** | 2.04 | (0.27, 19.54) | 0.497 |
| *OR adjusted for all covariates in the table  **^†^** Not including stroke | | | |

| **Table S7. Odds ratio (OR) and 95% confidence interval (CI) of SBP and potential confounders on the odds of severe COVID-19 amongst individuals with hypertension who are treated with monotherapy (N=1,798)** | | | |
| --- | --- | --- | --- |
|  | **OR*** | **95% CI** | **p-value** |
| **SBP (<120mmHg)** | 1.18 | (0.80, 1.74) | 0.407 |
| **Ref: SBP (120-129mmHg)** | 1 |  |  |
| **SBP (130-140mmHg)** | 1.15 | (0.85, 1.55) | 0.377 |
| **SBP (140-150mmHg)** | 1.16 | (0.83, 1.63) | 0.391 |
| **SBP (150-160mmHg)** | 2.03 | (1.29, 3.18) | 0.002 |
| **SBP (160-170mmHg)** | 1.67 | (0.92, 2.98) | 0.086 |
| **SBP (170-180mmHg)** | 1.84 | (0.79, 4.24) | 0.153 |
| **SBP (180+mmHg)** | 1.57 | (0.58, 4.06) | 0.358 |
| **Ref: SES (Least Deprived)** | 1 |  | 0.739 |
| **SES (Less Deprived)** | 0.94 | (0.67, 1.33) | 0.724 |
| **SES (More Deprived)** | 1.11 | (0.80, 1.55) | 0.546 |
| **SES (Most Deprived)** | 1.06 | (0.77, 1.46) | 0.716 |
| **Sex (Male)** | 1.33 | (1.07, 1.65) | 0.009 |
| **Ref: BMI (<25)** | 1 |  |  |
| **BMI (25-30)** | 1.04 | (0.77, 1.41) | 0.811 |
| **BMI (30-35)** | 1.14 | (0.82, 1.58) | 0.454 |
| **BMI (35+)** | 1.57 | (1.08, 2.29) | 0.018 |
| **CV Comorbidity^†^** | 1.19 | (0.95, 1.49) | 0.130 |
| **Stroke** | 2.37 | (1.49, 3.79) | <0.001 |
| **Diabetes** | 1.39 | (1.09, 1.76) | 0.007 |
| **Current Smoker** | 1.64 | (1.17, 2.29) | 0.004 |
| **Ethnicity (Non-white)** | 1.10 | (0.78, 1.53) | 0.577 |
| **Ref: Age (<60)** | 1 |  |  |
| **Age (60-70)** | 1.55 | (1.08, 2.26) | 0.020 |
| **Age (70-80)** | 3.80 | (2.67, 5.48) | <0.001 |
| **Age (80+)** | 8.74 | (5.34, 14.51) | <0.001 |
| **Ref: CRP (<3mg/L)** | 1 |  |  |
| **CRP (3-10mg/L)** | 1.12 | (0.88, 1.41) | 0.367 |
| **CRP (10-100mg/L)** | 1.04 | (0.70, 1.52) | 0.858 |
| **CRP (100+ mg/L)** | 2.33 | (0.26, 20.56) | 0.415 |

*OR adjusted for all covariates in the table

**^†^** Not including stroke

| **Table S8. Odds ratio (OR) and 95% confidence interval (CI) of SBP and potential confounders on the odds of severe COVID-19 amongst individuals with hypertension who are treated and where SBP is the mean SBP measured since 2016 (N=3,921)** | | | | |
| --- | --- | --- | --- | --- |
|  | **OR*** | **95% CI** | **p-value** |  |
| **SBP (<120mmHg)** | 1.30 | (0.95, 1.79) | 0.100 |  |
| **Ref: SBP (120-130mmHg)** | 1 |  |  |  |
| **SBP (130-140mmHg)** | 0.81 | (0.67, 0.99) | 0.037 |  |
| **SBP (140-150mmHg)** | 0.76 | (0.62, 0.95) | 0.014 |  |
| **SBP (150-160mmHg)** | 0.86 | (0.62, 1.18) | 0.344 |  |
| **SBP (160-170mmHg)** | 1.04 | (0.52, 2.01) | 0.900 |  |
| **SBP (170-180mmHg)** | 3.72 | (0.62, 22.36) | 0.135 |  |
| **SBP (180+mmHg)** | 0.48 | (0.02, 3.55) | 0.522 |  |
| **Ref: SES (Least Deprived)** | 1 |  |  |  |
| **SES (Less Deprived)** | 1.19 | (0.93, 1.51) | 0.163 |  |
| **SES (More Deprived)** | 1.31 | (1.04, 1.65) | 0.025 |  |
| **SES (Most Deprived)** | 1.46 | (1.17, 1.82) | 0.001 |  |
| **Sex (Male)** | 1.49 | (1.28, 1.73) | <0.001 |  |
| **Ref: BMI (<25)** | 1 |  |  |  |
| **BMI (25-30)** | 0.97 | (0.79, 1.20) | 0.787 |  |
| **BMI (30-35)** | 1.05 | (0.84, 1.32) | 0.647 |  |
| **BMI (35+)** | 1.64 | (1.27, 2.13) | <0.001 |  |
| **CV Comorbidity^†^** | 1.32 | (1.13, 1.53) | <0.001 |  |
| **Stroke** | 1.61 | (1.20, 2.15) | 0.001 |  |
| **Diabetes** | 1.56 | (1.33, 1.82) | <0.001 |  |
| **Current Smoker** | 1.62 | (1.28, 2.05) | <0.001 |  |
| **Ethnicity (Non-white)** | 1.18 | (0.92, 1.50) | 0.185 |  |
| **Ref: Age (<60)** | 1 |  |  |  |
| **Age (60-70)** | 1.97 | (1.47, 2.68) | <0.001 |  |
| **Age (70-80)** | 4.96 | (3.74, 6.66) | <0.001 |  |
| **Age (80+)** | 9.36 | (6.55, 13.49) | <0.001 |  |
| **Ref: CRP (<3mg/L)** | 1 |  |  |  |
| **CRP (3-10mg/L)** | 1.15 | (0.98, 1.36) | 0.090 |  |
| **CRP (10-100mg/L)** | 1.55 | (1.19, 2.00) | 0.001 |  |
| **CRP (100+ mg/L)** | 3.23 | (0.75, 14.72) | 0.113 |  |
| *OR adjusted for all covariates in the table  **^†^** Not including stroke | | | |  |

| **Table S9. Odds ratio (OR) and 95% confidence interval (CI) of SBP and potential confounders on the odds of severe COVID-19 amongst individuals with hypertension (N=6,517)** | | | |
| --- | --- | --- | --- |
| **Covariate** | **OR*** | **95% CI** | **p-value** |
| **Untreated SBP (<120mmHg)** | 1.64 | (1.14, 2.38) | 0.008 |
| **Ref: Untreated SBP (120-129mmHg)** | 1 | - | - |
| **Untreated SBP (130-139mmHg)** | 0.99 | (0.73, 1.33) | 0.934 |
| **Untreated SBP (140-149mmHg)** | 0.97 | (0.69, 1.36) | 0.859 |
| **Untreated SBP (150-159mmHg)** | 1.52 | (0.97, 2.34) | 0.062 |
| **Untreated SBP (160-169mmHg)** | 0.93 | (0.48, 1.71) | 0.824 |
| **Untreated SBP (170-179mmHg)** | 1.54 | (0.66, 3.42) | 0.298 |
| **Untreated SBP (180+mmHg)** | 0.79 | (0.25, 2.16) | 0.671 |
| **Treated SBP (<120mmHg)** | 1.55 | (1.15, 2.09) | 0.004 |
| **Treated SBP (120-129mmHg)** | 1.12 | (0.85, 1.48) | 0.418 |
| **Treated SBP (130-139mmHg)** | 1.16 | (0.90, 1.51) | 0.264 |
| **Treated SBP (140-149mmHg)** | 1.34 | (1.02, 1.77) | 0.039 |
| **Treated SBP (150-159mmHg)** | 2.18 | (1.56, 3.05) | <0.001 |
| **Treated SBP (160-169mmHg)** | 1.99 | (1.30, 3.03) | 0.001 |
| **Treated SBP (170-179mmHg)** | 2.15 | (1.19, 3.87) | 0.010 |
| **Treated SBP (180+mmHg)** | 2.17 | (1.16, 4.04) | 0.015 |
| **Ref: SES (Least Deprived)** | 1 | - | - |
| **SES (Less Deprived)** | 1.17 | (0.98, 1.41) | 0.088 |
| **SES (More Deprived)** | 1.32 | (1.10, 1.58) | 0.003 |
| **SES (Most Deprived)** | 1.36 | (1.15, 1.61) | <0.001 |
| **Diabetes** | 1.48 | (1.30, 1.68) | <0.001 |
| **Smoker** | 1.66 | (1.39, 1.97) | <0.001 |
| **Ethnicity (Non-White)** | 1.03 | (0.85, 1.25) | 0.755 |
| **Ref: Age (<60)** | 1 | - | - |
| **Age (60-70)** | 1.76 | (1.43, 2.17) | <0.001 |
| **Age (70-80)** | 4.26 | (3.50, 5.22) | <0.001 |
| **Age (80+)** | 7.70 | (5.91, 10.07) | <0.001 |
| **Ref: BMI (<25)** | 1 | - | - |
| **BMI (25-30)** | 1.04 | (0.88, 1.22) | 0.651 |
| **BMI (30-35)** | 1.12 | (0.94, 1.33) | 0.223 |
| **BMI (35+)** | 1.68 | (1.38, 2.05) | <0.001 |
| **CV Comorbidity^†^** | 1.42 | (1.26, 1.60) | <0.001 |
| **Stroke** | 1.43 | (1.12, 1.82) | 0.004 |
| **Sex (Male)** | 1.47 | (1.30, 1.65) | <0.001 |
| **Ref: CRP (<3 mg/L)** | 1 |  |  |
| **CRP (3-10 mg/L)** | 1.10 | (0.97, 1.25) | 0.132 |
| **CRP (10-100 mg/L)** | 1.57 | (1.28, 1.92) | <0.001 |
| **CRP (100+ mg/L)** | 1.94 | (0.53, 7.08) | 0.310 |
| *OR adjusted for all covariates in the table  **^†^** Not including stroke  The number of individuals in each category of SBP differed somewhat, the proportion of severe COVID-19 events in each category was greater in treated hypertensives: treated SBP<120 (n=537 (8%), events=217 (40%)), treated SBP:120-129 (n=933 (14%), events=287 (31%)), treated SBP:130-139 (n=1,483 (23%), events=459 (31%)), treated SBP:140-149 (n=869 (13%), events=296 (34%)), treated SBP:150-159 (n=307 (5%), events=135 (44%)), treated SBP:160-169 (n=149 (2%), events=65 (44%)), treated SBP:170-179 (n=61 (1%), events=29 (48%)), treated SBP:180+ (n=52 (1%), events=24 (46%)) and untreated SBP<120 (n=236 (4%), events=86 (36%)), untreated SBP:120-129 (n=459 (7%), events=107 (23%)), untreated SBP:130-139 (n=709 (11%), events=153 (22%)), untreated SBP:140-149 (n=423 (6%), events=92 (22%)), untreated SBP:150-159 (n=160 (2%), events=43 (27%)), untreated SBP:160-169 (n=80 (1%), events=16 (20%)), untreated SBP:170-179 (n=35 (1%), events=10 (29%)) and untreated SBP:180+ (n=24 (<1%), events=5 (21%)). | | | |

| **Table S10. Odds ratio (OR) and 95% confidence interval (CI) of DBP and potential confounders on the odds of severe COVID-19 amongst individuals with hypertension who are treated (N=4,391)** | | | | |
| --- | --- | --- | --- | --- |
| Covariate | **OR*** | **95% CI** | **p-value** |  |
| **DBP (<60mmHg)** | 1.37 | (0.89, 2.10) | 0.149 |  |
| **DBP (60-70mmHg)** | 0.97 | (0.79, 1.19) | 0.771 |  |
| **DBP (70-80mmHg)** | 0.86 | (0.73, 1.02) | 0.081 |  |
| **Ref: DBP (80-90mmHg)** | 1 | - | - |  |
| **DBP (90-100mmHg)** | 1.43 | (1.11, 1.83) | 0.005 |  |
| **DBP (100+mmHg)** | 1.37 | (0.89, 2.10) | <0.001 |  |
| **Ref: SES (Least Deprived)** | 1 |  |  |  |
| **SES (Less Deprived)** | 1.16 | (0.93, 1.44) | 0.189 |  |
| **SES (More Deprived)** | 1.29 | (1.04, 1.59) | 0.019 |  |
| **SES (Most Deprived)** | 1.37 | (1.12, 1.67) | 0.002 |  |
| **Sex (Male)** | 1.48 | (1.29, 1.70) | <0.001 |  |
| **Ref: BMI (<25)** | 1 |  |  |  |
| **BMI (25-30)** | 1.08 | (0.89, 1.31) | 0.431 |  |
| **BMI (30-35)** | 1.10 | (0.89, 1.36) | 0.381 |  |
| **BMI (35+)** | 1.76 | (1.39, 2.23) | <0.001 |  |
| **CV Comorbidity^†^** | 1.29 | (1.12, 1.48) | <0.001 |  |
| **Stroke** | 1.54 | (1.16, 2.03) | 0.002 |  |
| **Diabetes** | 1.46 | (1.26, 1.69) | <0.001 |  |
| **Current Smoker** | 1.60 | (1.30, 1.98) | <0.001 |  |
| **Ethnicity (Non-white)** | 1.07 | (0.86, 1.34) | 0.547 |  |
| **Ref: Age (<60)** | 1 |  |  |  |
| **Age (60-70)** | 1.83 | (1.42, 2.39) | <0.001 |  |
| **Age (70-80)** | 4.59 | (3.57, 5.95) | <0.001 |  |
| **Age (80+)** | 8.50 | (6.15, 11.82) | <0.001 |  |
| **Ref: CRP (<3mg/L)** | 1 |  |  |  |
| **CRP (3-10mg/L)** | 1.08 | (0.93, 1.26) | 0.308 |  |
| **CRP (10-100mg/L)** | 1.46 | (1.15, 1.85) | 0.002 |  |
| **CRP (100+ mg/L)** | 2.42 | (0.58, 10.65) | 0.222 |  |
| *OR adjusted for all covariates in the table  **^†^** Not including stroke | | | |  |

| **Table S11. Odds ratio (OR) and 95% confidence interval (CI) for the association between ACEi and ARB antihypertensive medications and the odds of severe COVID-19, compared to ‘other’ antihypertensive medication amongst individuals with hypertension who were treated* (N=4,362)** | | | | | |
| --- | --- | --- | --- | --- | --- |
| **Covariate** | **OR^†^** | **95% CI** | **p-value** |  |  |
| **Ref: Med (Other)^‡^** | 1 | - | - |  |  |
| **Med (ACEi)** | 0.96 | (0.83, 1.12) | 0.623 |  |  |
| **Med (ARB)** | 1.11 | (0.92, 1.34) | 0.254 |  |  |
| **SBP (<120mmHg)** | 1.39 | (1.09, 1.76) | 0.007 |  |  |
| **Ref: SBP (120-130mmHg)** | 1 | - | - |  |  |
| **SBP (130-140mmHg)** | 1.02 | (0.84, 1.23) | 0.849 |  |  |
| **SBP (140-150mmHg)** | 1.18 | (0.95, 1.45) | 0.126 |  |  |
| **SBP (150-160mmHg)** | 1.9 | (1.43, 2.52) | <0.001 |  |  |
| **SBP (160+mmHg)** | 1.8 | (1.33, 2.43) | <0.001 |  |  |
| **Ref: SES (Least Deprived)** | 1 | - | - |  |  |
| **SES (Less Deprived)** | 1.13 | (0.91, 1.41) | 0.263 |  |  |
| **SES (More Deprived)** | 1.26 | (1.02, 1.56) | 0.031 |  |  |
| **SES (Most Deprived)** | 1.33 | (1.09, 1.63) | 0.005 |  |  |
| **Sex (Male)** | 1.49 | (1.3, 1.71) | <0.001 |  |  |
| **Diabetes** | 1.42 | (1.22, 1.64) | <0.001 |  |  |
| **Ethnicity (Non-White)** | 1.07 | (0.86, 1.34) | 0.529 |  |  |
| **Ref: Age (<60)** | 1 | - | - |  |  |
| **Age (60-70)** | 1.73 | (1.34, 2.25) | <0.001 |  |  |
| **Age (70-80)** | 4.1 | (3.21, 5.29) | <0.001 |  |  |
| **Age (80+)** | 7.48 | (5.44, 10.36) | <0.001 |  |  |
| **CV Comorbidity^§^** | 1.28 | (1.12, 1.48) | <0.001 |  |  |
| **Stroke** | 1.52 | (1.15, 2) | 0.003 |  |  |
| **Smoker** | 1.67 | (1.35, 2.07) | <0.001 |  |  |
| **Ref: BMI (<25)** | 1 | - | - |  |  |
| **BMI (25-30)** | 1.07 | (0.89, 1.31) | 0.47 |  |  |
| **BMI (30-35)** | 1.12 | (0.91, 1.39) | 0.285 |  |  |
| **BMI (35+)** | 1.75 | (1.38, 2.22) | <0.001 |  |  |
| **Ref: CRP (<3 mg/L)** | 1 |  |  |  |  |
| **CRP (3-10 mg/L)** | 1.45 | (1.14, 1.85) | 0.002 |  |  |
| **CRP (10-100 mg/L)** | 2.19 | (0.5, 10.15) | 0.296 |  |  |
| **CRP (100+ mg/L)** | 1.1 | (0.94, 1.27) | 0.235 |  |  |
| *Individuals who were taking both ACEi & ARBs were excluded from these analyses  **^†^**Adjusted OR (odds ratio), model adjusted for all covariates in the table  **^‡^**Chi-squared test of medication (ACEi, ARB or other) and severe COVID-19: chi-squared = 1.22, p-value=0.544  **^§^**Not including stroke | | | | | |

| **Table S12. Association between ACEi and ARB antihypertensive medications and the odds of severe COVID-19, compared to ‘other’ antihypertensive medication amongst individuals with hypertension who were treated with combination therapy (N=2,564)** | | | | | |
| --- | --- | --- | --- | --- | --- |
| **Covariate** | **OR^*^** | **95% CI** | **p-value** |  |  |
| **Ref: Med (Other)** | 1 | - | - |  |  |
| **Med (ACEi)** | 1.01 | (0.82, 1.25) | 0.932 |  |  |
| **Med (ARB)** | 1.12 | (0.88, 1.42) | 0.378 |  |  |
| **SBP (<120mmHg)** | 1.53 | (1.13, 2.07) | 0.005 |  |  |
| **Ref: SBP (120-130mmHg)** | 1 | - | - |  |  |
| **SBP (130-140mmHg)** | 0.94 | (0.74, 1.20) | 0.616 |  |  |
| **SBP (140-150mmHg)** | 1.18 | (0.90, 1.55) | 0.220 |  |  |
| **SBP (150-160mmHg)** | 1.81 | (1.25, 2.62) | 0.002 |  |  |
| **SBP (160-170mmHg)** | 1.86 | (1.12, 3.07) | 0.015 |  |  |
| **SBP (170-180mmHg)** | 1.86 | (0.87, 3.97) | 0.107 |  |  |
| **SBP (180+mmHg)** | 2.22 | (1.02, 4.85) | 0.043 |  |  |
| **Ref: SES (Least Deprived)** | 1 | - | - |  |  |
| **SES (Less Deprived)** | 1.26 | (0.95, 1.67) | 0.114 |  |  |
| **SES (More Deprived)** | 1.37 | (1.04, 1.81) | 0.024 |  |  |
| **SES (Most Deprived)** | 1.54 | (1.19, 2.00) | 0.001 |  |  |
| **Sex (Male)** | 1.62 | (1.35, 1.96) | <0.001 |  |  |
| **Diabetes** | 1.42 | (1.18, 1.72) | <0.001 |  |  |
| **Ethnicity (Non-White)** | 1.10 | (0.81, 1.49) | 0.537 |  |  |
| **Ref: Age (<60)** | 1 | - | - |  |  |
| **Age (60-70)** | 1.94 | (1.35, 2.83) | <0.001 |  |  |
| **Age (70-80)** | 4.55 | (3.22, 6.54) | <0.001 |  |  |
| **Age (80+)** | 7.39 | (4.82, 11.5) | <0.001 |  |  |
| **CV Comorbidity ^†^** | 1.34 | (1.11, 1.60) | 0.002 |  |  |
| **Stroke** | 1.21 | (0.85, 1.72) | 0.288 |  |  |
| **Smoker** | 1.71 | (1.30, 2.25) | <0.001 |  |  |
| **Ref: BMI (<25)** | 1 | - | - |  |  |
| **BMI (25-30)** | 1.11 | (0.86, 1.43) | 0.443 |  |  |
| **BMI (30-35)** | 1.12 | (0.85, 1.48) | 0.420 |  |  |
| **BMI (35+)** | 1.90 | (1.39, 2.61) | <0.001 |  |  |
| **Ref: CRP (<3 mg/L)** | 1 |  |  |  |  |
| **CRP (3-10 mg/L)** | 1.09 | (0.89, 1.32) | 0.410 |  |  |
| **CRP (10-100 mg/L)** | 1.81 | (1.32, 2.48) | <0.001 |  |  |
| **CRP (100+ mg/L)** | 2.04 | (0.27, 19.63) | 0.499 |  |  |
| **^*^**Adjusted OR (odds ratio), model adjusted for all covariates in the table  **^†^**Not including stroke | | | | | |

| **Table S13. Odds ratio (OR) and 95% confidence interval (CI) for the association between ACEi and ARB antihypertensive medications and the odds of severe COVID-19, compared to ‘other’ antihypertensive medication amongst individuals with hypertension who were treated with monotherapy (N=1,798)** | | | | |
| --- | --- | --- | --- | --- |
| **Covariate** | **OR^*^** | **95% CI** | **p-value** |  |
| **Ref: Med (Other)** | 1 | - | - |  |
| **Med (ACEi)** | 0.91 | (0.69, 1.21) | 0.525 |  |
| **Med (ARB)** | 1.25 | (0.85, 1.82) | 0.244 |  |
| **SBP (<120mmHg)** | 1.18 | (0.80, 1.75) | 0.402 |  |
| **Ref: SBP (120-130mmHg)** | 1 | - | - |  |
| **SBP (130-140mmHg)** | 1.14 | (0.85, 1.54) | 0.391 |  |
| **SBP (140-150mmHg)** | 1.16 | (0.83, 1.63) | 0.393 |  |
| **SBP (150-160mmHg)** | 2.03 | (1.29, 3.18) | 0.002 |  |
| **SBP (160-170mmHg)** | 1.65 | (0.91, 2.96) | 0.091 |  |
| **SBP (170-180mmHg)** | 1.83 | (0.79, 4.24) | 0.156 |  |
| **SBP (180+mmHg)** | 1.53 | (0.57, 3.95) | 0.383 |  |
| **Ref: SES (Least Deprived)** | 1 | - | - |  |
| **SES (Less Deprived)** | 0.94 | (0.66, 1.33) | 0.715 |  |
| **SES (More Deprived)** | 1.12 | (0.80, 1.56) | 0.519 |  |
| **SES (Most Deprived)** | 1.06 | (0.78, 1.46) | 0.699 |  |
| **Sex (Male)** | 1.35 | (1.09, 1.68) | 0.007 |  |
| **Diabetes** | 1.39 | (1.09, 1.77) | 0.007 |  |
| **Ethnicity (Non-White)** | 1.08 | (0.77, 1.50) | 0.657 |  |
| **Ref: Age (<60)** | 1 | - | - |  |
| **Age (60-70)** | 1.54 | (1.07, 2.25) | 0.022 |  |
| **Age (70-80)** | 3.78 | (2.65, 5.46) | <0.001 |  |
| **Age (80+)** | 8.64 | (5.26, 14.37) | <0.001 |  |
| **CV Comorbidity ^†^** | 1.19 | (0.95, 1.50) | 0.130 |  |
| **Stroke** | 2.34 | (1.47, 3.76) | <0.001 |  |
| **Smoker** | 1.66 | (1.18, 2.32) | 0.003 |  |
| **Ref: BMI (<25)** | 1 | - | - |  |
| **BMI (25-30)** | 1.03 | (0.76, 1.39) | 0.865 |  |
| **BMI (30-35)** | 1.12 | (0.80, 1.57) | 0.498 |  |
| **BMI (35+)** | 1.56 | (1.07, 2.27) | 0.020 |  |
| **Ref: CRP (<3 mg/L)** | 1 | - | - |  |
| **CRP (3-10 mg/L)** | 1.11 | (0.87, 1.41) | 0.393 |  |
| **CRP (10-100 mg/L)** | 1.03 | (0.70, 1.51) | 0.870 |  |
| **CRP (100+ mg/L)** | 2.37 | (0.26, 21.05) | 0.409 |  |
| **^*^**Adjusted OR (odds ratio), model adjusted for all covariates in the table  **^†^**Not including stroke | | | | |

| **Table S14. Odds ratio (OR) and 95% confidence interval (CI) for the association between ACEi and ARB antihypertensive medications and the odds of severe COVID-19, compared to ‘other’ antihypertensive medication amongst individuals with hypertension who were treated, where systolic SBP is the mean SBP measured since 2016 (N=3,921)** | | | | |
| --- | --- | --- | --- | --- |
| **Covariate** | **OR^*^** | **95% CI** | **p-value** |  |
| **Ref: Med (Other)** | 1 | - | - |  |
| **Med (ACEi)** | 0.88 | (0.74, 1.03) | 0.120 |  |
| **Med (ARB)** | 0.98 | (0.80, 1.20) | 0.853 |  |
| **SBP (<120mmHg)** | 0.76 | (0.56, 1.05) | 0.095 |  |
| **Ref: SBP (120-130mmHg)** | 1 | - | - |  |
| **SBP (130-140mmHg)** | 0.62 | (0.46, 0.84) | 0.002 |  |
| **SBP (140-150mmHg)** | 0.58 | (0.43, 0.80) | 0.001 |  |
| **SBP (150-160mmHg)** | 0.65 | (0.44, 0.97) | 0.035 |  |
| **SBP (160-170mmHg)** | 0.80 | (0.38, 1.60) | 0.527 |  |
| **SBP (170-180mmHg)** | 2.77 | (0.45, 16.88) | 0.251 |  |
| **SBP (180+mmHg)** | 0.37 | (0.02, 2.76) | 0.387 |  |
| **Ref: SES (Least Deprived)** | 1 | - | - |  |
| **SES (Less Deprived)** | 1.18 | (0.93, 1.51) | 0.174 |  |
| **SES (More Deprived)** | 1.30 | (1.03, 1.64) | 0.028 |  |
| **SES (Most Deprived)** | 1.45 | (1.17, 1.81) | 0.001 |  |
| **Sex (Male)** | 1.51 | (1.29, 1.76) | <0.001 |  |
| **Diabetes** | 1.57 | (1.34, 1.84) | <0.001 |  |
| **Ethnicity (Non-White)** | 1.16 | (0.91, 1.48) | 0.228 |  |
| **Ref: Age (<60)** | 1 | - | - |  |
| **Age (60-70)** | 1.96 | (1.46, 2.66) | <0.001 |  |
| **Age (70-80)** | 4.91 | (3.70, 6.60) | <0.001 |  |
| **Age (80+)** | 9.21 | (6.44, 13.3) | <0.001 |  |
| **CV Comorbidity ^†^** | 1.31 | (1.13, 1.53) | <0.001 |  |
| **Stroke** | 1.61 | (1.20, 2.15) | 0.001 |  |
| **Smoker** | 1.63 | (1.29, 2.05) | <0.001 |  |
| **Ref: BMI (<25)** | 1 | - | - |  |
| **BMI (25-30)** | 0.97 | (0.79, 1.20) | 0.799 |  |
| **BMI (30-35)** | 1.05 | (0.84, 1.32) | 0.662 |  |
| **BMI (35+)** | 1.64 | (1.27, 2.12) | <0.001 |  |
| **Ref: CRP (<3 mg/L)** | 1 | - | - |  |
| **CRP (3-10 mg/L)** | 1.15 | (0.98, 1.36) | 0.092 |  |
| **CRP (10-100 mg/L)** | 1.54 | (1.19, 1.99) | 0.001 |  |
| **CRP (100+ mg/L)** | 3.29 | (0.76, 15.06) | 0.109 |  |
| **^*^**Adjusted OR (odds ratio), model adjusted for all covariates in the table  **^†^**Not including stroke | | | | |

| **Table S15. Hazard ratio’s (HR) and 95% confidence intervals (CI) of SBP and potential confounders (N=4,391) and Antihypertensive medications and potential confounders (N=4,362)* on the time to severe COVID-19 in treated individuals with essential (primary) hypertension** | | | | | | |  |
| --- | --- | --- | --- | --- | --- | --- | --- |
|  | **SBP** | | | **Antihypertensive medications** | | | |
| **Covariate** | **HR**^†^ | **95% CI** | **p-value** | **HR**^†^ | **95% CI** | **p-value** | |
| **Ref: Med (Other)** | - | - | - | 1 | - | - | |
| **Med (ACEi)** | - | - | - | 0.99 | (0.88, 1.11) | 0.814 | |
| **Med (ARB)** | - | - | - | 1.07 | (0.93, 1.23) | 0.330 | |
| **SBP (<120mmHg)** | 1.30 | (1.09, 1.55) | 0.004 | 1.28 | (1.07, 1.53) | 0.007 | |
| **Ref: SBP (120-130mmHg)** | 1 | - | - | 1 | - | - | |
| **SBP (130-140mmHg)** | 1.02 | (0.88, 1.19) | 0.748 | 1.01 | (0.87, 1.18) | 0.864 | |
| **SBP (140-150mmHg)** | 1.12 | (0.95, 1.32) | 0.170 | 1.12 | (0.95, 1.31) | 0.185 | |
| **SBP (150-160mmHg)** | 1.69 | (1.37, 2.08) | <0.001 | 1.68 | (1.37, 2.06) | <0.001 | |
| **SBP (160-170mmHg)** | 1.52 | (1.16, 2.00) | 0.002 | 1.49 | (1.14, 1.96) | 0.004 | |
| **SBP (170-180mmHg)** | 1.62 | (1.1, 2.38) | 0.014 | 1.60 | (1.09, 2.35) | 0.016 | |
| **SBP (180+mmHg)** | 1.89 | (1.24, 2.87) | 0.003 | 1.87 | (1.23, 2.85) | 0.003 | |
| **Ref: SES (Least Deprived)** | 1 | - | - | 1 | - | - | |
| **SES (Less Deprived)** | 1.10 | (0.93, 1.31) | 0.260 | 1.11 | (0.94, 1.32) | 0.234 | |
| **SES (More Deprived)** | 1.19 | (1.01, 1.40) | 0.043 | 1.19 | (1.01, 1.41) | 0.035 | |
| **SES (Most Deprived)** | 1.26 | (1.08, 1.47) | 0.003 | 1.27 | (1.08, 1.48) | 0.003 | |
| **Ref: Age (<60)** | 1 | - | - | 1 | - | - | |
| **Age (60-70)** | 1.67 | (1.33, 2.10) | <0.001 | 1.66 | (1.32, 2.10) | <0.001 | |
| **Age (70-80)** | 3.36 | (2.70, 4.18) | <0.001 | 3.31 | (2.66, 4.11) | <0.001 | |
| **Age (80+)** | 5.13 | (3.98, 6.60) | <0.001 | 5.09 | (3.95, 6.56) | <0.001 | |
| **Smoker** | 1.53 | (1.32, 1.77) | <0.001 | 1.51 | (1.29, 1.75) | <0.001 | |
| **Ref: BMI (<25)** | 1 | - | - | 1 | - | - | |
| **BMI (25-30)** | 1.09 | (0.93, 1.26) | 0.286 | 1.08 | (0.93, 1.25) | 0.328 | |
| **BMI (30-35)** | 1.12 | (0.96, 1.32) | 0.159 | 1.12 | (0.95, 1.32) | 0.184 | |
| **BMI (35+)** | 1.58 | (1.32, 1.89) | <0.001 | 1.57 | (1.31, 1.88) | <0.001 | |
| **Diabetes** | 1.29 | (1.16, 1.44) | <0.001 | 1.29 | (1.16, 1.44) | <0.001 | |
| **Ethnicity (Non-white)** | 1.10 | (0.92, 1.31) | 0.279 | 1.09 | (0.92, 1.30) | 0.318 | |
| **CV Comorbidity^‡^** | 1.20 | (1.08, 1.33) | 0.001 | 1.21 | (1.09, 1.35) | 0.001 | |
| **Stroke** | 1.29 | (1.07, 1.56) | 0.008 | 1.29 | (1.06, 1.55) | 0.009 | |
| **Sex (Male)** | 1.38 | (1.24, 1.53) | <0.001 | 1.38 | (1.23, 1.53) | <0.001 | |
| **REF: CRP (<3 mg/L)** | 1 | - | - | 1 | - | - | |
| **CRP (3-10 mg/L)** | 1.06 | (0.95, 1.19) | 0.282 | 1.07 | (0.95, 1.20) | 0.265 | |
| **CRP (10-100 mg/L)** | 1.30 | (1.10, 1.55) | 0.003 | 1.30 | (1.10, 1.55) | 0.003 | |
| **CRP (100+ mg/L)** | 1.24 | (0.51, 3.00) | 0.639 | 1.25 | (0.51, 3.03) | 0.627 | |
| *Individuals taking both ACEi & ARBs were removed from analyses  ^†^HR’s adjusted for all covariates in the table  **^‡^**Not including stroke | | | | | | |  |

**Additional analyses: Updated definition of hypertension: ICD-10 ‘Essential (Primary) Hypertension (I10) or SBP ≥140mmHg or DBP ≥90mmHg**

| **Table S16. Odds ratio (OR) and 95% confidence intervals (CI) for the association of essential (primary) hypertension or hypertension defined by SBP ≥140mmHg or DBP ≥90mmHg and the odds of severe COVID-19 adjusting for: 1) potential confounding variables and 2) potential confounding variables and intermediate variables (N=17,094)** | | | | | | | |
| --- | --- | --- | --- | --- | --- | --- | --- |
|  | **Model adjusted for confounding variables** | | | **Model adjusted for confounding and intermediate variables** | | | |
| **Covariate** | **OR*** | **95% CI** | **p-value** | | **OR*** | **95% CI** | **p-value** |
| **Hypertension** | 1.38 | (1.26, 1.51) | <0.001 | | 1.33 | (1.22, 1.46) | <0.001 |
| **Ref: SES (Least Deprived)** | 1 | - | - | | 1 | - | - |
| **SES (Less Deprived)** | 1.03 | (0.91, 1.17) | 0.650 | | 1.02 | (0.90, 1.16) | 0.719 |
| **SES (More Deprived)** | 1.14 | (1.01, 1.29) | 0.032 | | 1.13 | (1.00, 1.28) | 0.046 |
| **SES (Most Deprived)** | 1.21 | (1.07, 1.36) | 0.002 | | 1.19 | (1.06, 1.34) | 0.004 |
| **Sex (Male)** | 1.45 | (1.34, 1.57) | <0.001 | | 1.41 | (1.30, 1.53) | <0.001 |
| **Ref: BMI (<25)** | 1 | - | - | | 1 | - | - |
| **BMI (25-30)** | 1.10 | (1.00, 1.22) | 0.061 | | 1.11 | (1.00, 1.23) | 0.050 |
| **BMI (30-35)** | 1.23 | (1.09, 1.39) | 0.001 | | 1.24 | (1.10, 1.40) | 0.001 |
| **BMI (35+)** | 1.77 | (1.53, 2.05) | <0.001 | | 1.76 | (1.52, 2.05) | <0.001 |
| **Diabetes** | 1.61 | (1.45, 1.79) | <0.001 | | 1.54 | (1.38, 1.71) | <0.001 |
| **Smoker** | 1.50 | (1.33, 1.70) | <0.001 | | 1.48 | (1.31, 1.67) | <0.001 |
| **Ethnicity (Non-White)** | 0.98 | (0.84, 1.13) | 0.745 | | 0.99 | (0.85, 1.14) | 0.851 |
| **Ref: Age (<60)** | 1 | - | - | | 1 | - | - |
| **Age (60-70)** | 1.61 | (1.42, 1.81) | <0.001 | | 1.57 | (1.39, 1.77) | <0.001 |
| **Age (70-80)** | 4.71 | (4.20, 5.28) | <0.001 | | 4.39 | (3.91, 4.93) | <0.001 |
| **Age (80+)** | 9.24 | (7.71, 11.08) | <0.001 | | 8.32 | (6.92, 10.01) | <0.001 |
| **Ref: CRP (3 mg/L)** | 1 |  |  | | 1 |  |  |
| **CRP (3-10 mg/L)** | 1.18 | (1.08, 1.30) | <0.001 | | 1.17 | (1.07, 1.29) | 0.001 |
| **CRP (10-100 mg/L)** | 1.57 | (1.35, 1.83) | <0.001 | | 1.53 | (1.31, 1.79) | <0.001 |
| **CRP (100+ mg/L)** | 3.01 | (1.32, 6.78) | 0.008 | | 2.93 | (1.28, 6.59) | 0.010 |
| **CV Comorbidity** | - | - | - | | 1.30 | (1.18, 1.42) | <0.001 |
| **Stroke** | - | - | - | | 1.46 | (1.17, 1.82) | 0.001 |
| *OR adjusted for covariates listed in the table. | | | | | | | |

| **Table S17. Odds ratio (OR) and 95% confidence intervals (CI) for SBP and potential confounders (N=4,747) and antihypertensive medications and potential confounders (N=4,718)* on the odds of severe COVID-19 amongst treated individuals with hypertension defined by ICD10 codes or by SBP ≥140mmHg or DBP ≥90mmHg** | | | | | | |  |  |  |
| --- | --- | --- | --- | --- | --- | --- | --- | --- | --- |
|  | **SBP** | | | **Antihypertensive**  **medications** | | | | |  |
| **Covariate** | **OR^†^** | **95% CI** | **p-value** | **OR^†^** | **95% CI** | **p-value** | |  |  |
| **Ref: Med (Other)** | - | - | - | 1 | - | - | |  |  |
| **Med (ACEi)** | - | - | - | 0.97 | (0.84, 1.13) | 0.712 | |  |  |
| **Med (ARB)** | - | - | - | 1.11 | (0.93, 1.34) | 0.243 | |  |  |
| **SBP (<120mmHg)** | 1.39 | (1.10, 1.76) | 0.007 | 1.37 | (1.08, 1.73) | 0.009 | |  |  |
| **Ref: SBP (120-130mmHg)** | 1 | - | - | 1 | - | - | |  |  |
| **SBP (130-140mmHg)** | 1.02 | (0.85, 1.23) | 0.842 | 1.01 | (0.84, 1.22) | 0.938 | |  |  |
| **SBP (140-150mmHg)** | 1.22 | (1.00, 1.49) | 0.055 | 1.21 | (0.99, 1.48) | 0.060 | |  |  |
| **SBP (150-160mmHg)** | 1.70 | (1.30, 2.23) | <0.001 | 1.70 | (1.29, 2.22) | <0.001 | |  |  |
| **SBP (160-170mmHg)^‡^** | 1.78 | (1.25, 2.51) | 0.001 | 1.88 | (1.42, 2.49) | <0.001 | |  |  |
| **SBP (170-180mmHg)** | 2.06 | (1.23, 3.44) | 0.006 | - | - | - | |  |  |
| **SBP (180mmHg+)** | 2.15 | (1.22, 3.80) | 0.008 | - | - | - | |  |  |
| **Ref: SES (Least Deprived)** | 1 | - | - | 1 | - | - | |  |  |
| **SES (Less Deprived)** | 1.11 | (0.90, 1.37) | 0.310 | 1.12 | (0.91, 1.38) | 0.292 | |  |  |
| **SES (More Deprived)** | 1.20 | (0.98, 1.47) | 0.074 | 1.21 | (0.99, 1.48) | 0.066 | |  |  |
| **SES (Most Deprived)** | 1.32 | (1.09, 1.60) | 0.005 | 1.32 | (1.09, 1.61) | 0.004 | |  |  |
| **Sex (Male)** | 1.47 | (1.29, 1.68) | <0.001 | 1.47 | (1.29, 1.69) | <0.001 | |  |  |
| **Ref: BMI (<25)** | 1 | - | - | 1 | - | - | |  |  |
| **BMI (25-30)** | 1.07 | (0.89, 1.29) | 0.466 | 1.06 | (0.88, 1.28) | 0.52 | |  |  |
| **BMI (30-35)** | 1.17 | (0.96, 1.43) | 0.129 | 1.16 | (0.95, 1.42) | 0.157 | |  |  |
| **BMI (35+)** | 1.75 | (1.39, 2.20) | <0.001 | 1.72 | (1.37, 2.17) | <0.001 | |  |  |
| **Diabetes** | 1.39 | (1.20, 1.60) | <0.001 | 1.39 | (1.20, 1.60) | <0.001 | |  |  |
| **Smoker** | 1.66 | (1.36, 2.04) | <0.001 | 1.67 | (1.36, 2.05) | <0.001 | |  |  |
| **Ethnicity (Non-White)** | 1.06 | (0.85, 1.32) | 0.593 | 1.05 | (0.84, 1.30) | 0.681 | |  |  |
| **Ref: Age (<60)** | 1 | - | - | 1 | - | - | |  |  |
| **Age (60-70)** | 1.75 | (1.37, 2.24) | <0.001 | 1.74 | (1.36, 2.23) | <0.001 | |  |  |
| **Age (70-80)** | 4.27 | (3.39, 5.43) | <0.001 | 4.21 | (3.34, 5.35) | <0.001 | |  |  |
| **Age (80+)** | 7.96 | (5.88, 10.85) | <0.001 | 7.94 | (5.86, 10.81) | <0.001 | |  |  |
| **Ref: CRP (<3 mg/L)** | 1 |  |  | 1 |  |  | |  |  |
| **CRP (3-10 mg/L)** | 1.10 | (0.95, 1.27) | 0.209 | 1.10 | (0.95, 1.27) | 0.185 | |  |  |
| **CRP (10-100 mg/L)** | 1.48 | (1.18, 1.87) | 0.001 | 1.49 | (1.18, 1.88) | 0.001 | |  |  |
| **CRP (100+ mg/L)** | 2.25 | (0.52, 10.28) | 0.276 | 2.27 | (0.52, 10.39) | 0.272 | |  |  |
| **CV Comorbidity^§^** | 1.27 | (1.10, 1.45) | 0.001 | 1.28 | (1.11, 1.46) | <0.001 | |  |  |
| **Stroke** | 1.56 | (1.18, 2.05) | 0.002 | 1.54 | (1.17, 2.03) | 0.002 | |  |  |
| *Individuals taking both ACEi and ARB’s were removed from these analyses  **^†^**OR adjusted for all covariates listed in the table  ^‡^SBP (160+mmHg) for antihypertensive medications model  **^§^**Not including stroke | | | | | | | | | |

**Methods Supplement**

**Medications/Prescriptions**

| **Table S18. BNF paragraphs, products, chemical substances and presentation codes for medications** | | | |
| --- | --- | --- | --- |
| **BNF Paragraphs** | | | |
| **Antihypertensive drugs** | | **Anticoagulants** | |
| Thiazides And Related Diuretics | | Oral Anticoagulants | |
| Pot-Sparing Diuretics&Aldosterone Antag | | Parental Anticoagulants | |
| Potassium Sparing Diuretics & Compounds | |  | |
| Diuretics With Potassium | |  | |
| Beta-Adrenoceptor Blocking Drugs | |  | |
| Vasodilator Antihypertensive Drugs | |  | |
| Centrally-Acting Antihypertensive Drugs | |  | |
| Renin-Angiotensin System Drugs | |  | |
|  | | | |
| **BNF Product/BNF chemical substance/BNF presentation** | | | |
| **Statins** | **ACEi** | | **ARBs** |
| atorvastatin | benazepril | | eprosartan |
| Lipitor | lotensin | | olmesartan |
| rosuvastatin | captopril | | valsartan |
| crestor | enalapril | | candesartan |
| simvastatin | vasotec | | losartan |
| Zocor | enalaprilat | | telmisartan |
| fluvastatin | fosinopril | | irbesartan |
| lescol | Lisinopril | | azilsartan |
| pravastatin | Zestril | | valsartan |
| lipostat | prinivil | |  |
|  | moexipril | |  |
|  | perindopril | |  |
|  | quinapril | |  |
|  | accupril | |  |
|  | Ramipril | |  |
|  | altace | |  |
|  | trandolapril | |  |
|  | cilazapril | |  |
|  | imidapril | |  |
|  | vascace | |  |
|  | tanatril | |  |
|  |  | |  |
| **BNF Codes** | | | |
|  | **Code** | | |
| **ARB** | 0205052 | | |
| **ACEi** | 0205051 | | |
| **Other RAS drugs** | 0205053A0AA, 0205053A0BB | | |
| **Thiazides** | 020201 | | |
| **Pot-sparing diuretics** | 020201 | | |
| **Diuretic with potassium** | 020208 | | |
| **Beta-blockers** | 020400 (excluding: 0204000L, 0204000M, 0204000N, 0204000P, 0204000T, 0204000V, 0204000X, 0204000Y, 0204000Z) | | |
| **Vasodilator antihypertensive drugs** | 020501 (excluding: 0205010A, 0205010U, 0205010V, 0205010W, 0205010X, 0205010Y, 0205010Z) | | |
| **Centrally acting antihypertensives** | 020502 (excluding: 020502000B, 0205020G, 0205020N, 0205020P, 0205020Q, 0205020R) | | |
| **Renin-angiotensin system drugs** | 0205053 (excluding: ARBs & ACEi) | | |
| **Alpha-blockers** | 020504 (excluding: 0205040K) | | |
| **Nitrates** | 020601 (excluding: 0206010F, 0206010A, 0206010V) | | |
| **Calcium-channel blockers** | 020602 | | |
| **Anticoagulants** | 020801, 020802 | | |
| **Statins** | 0212000B0AA, 0212000B0BB, 0212000AAAA, 0212000AABB, 0212000AJAA, 0212000Y0AA, 0212000Y0BF, 0212000Y0BB, 0212000M0AA,0212000M0BB, 0212000M0BC, 0212000M0BD, 0212000M0BE, 0212000M0BF, 0212000M0BG, 0212000M0BB, 0212000X0AA, 0212000X0BB, 0212000X0BC, 0212000X0BD, 0212000X0BE, 0212000X0BB | | |
| Antihypertensive drugs are classified as ‘other’ if they are defined as Antihypertensive drugs (above), and are not ARB or ACEi based on drug names (above). | | | |

| **Table S19. Names of drugs included for each medication class** | |
| --- | --- |
| **Drugs list** |  |
| **ARB** | Irbesartan/Hydchloroth, CoAprovel, Olmesartan Medoxomil/Amlodipine, Sevikar, Olmesartan Medox/Amlodipine/Hydchloroth, Sevikar HCT, Azilsartan Medoxomil, Edarbi, Sacubitril/Valsartan, Entresto, Olmesartan Medoxomil, Olmetec, Candesartan Cilexetil, Amias, Atacand, Ratacand, Irbesartan, Aprovel, Karvea, Sabervel, Ifirmasta, Losartan Pot, Cozaar, Losaprex, Losartam Pot/Hydchloroth, Cozaar-Comp, Hyzaar, Telmisartan, Micardis, Tolura, Telmisartan/Hydchloroth, MicardisPlus, Actelsar, Tolucombi, Valsartan, Diovan, Eprosartan, Tevetn, Valsartan/HydchlorothCo-Diovan, Olmesartan Medoxomil/Hydchloroth, Olmetec Plus |
| **ACEi** | Perindopril Tosilate, Perindopril Tosilate/Indapamide, Perindopril Erbumine + Amlodipine, Moexipril HCL, Perdix, Cilazapril, Vascace, Captopril, Acepril, Capoten, Ecopace, Tensopril, Kaplon, Hyteneze, Captomex, Noyada, Co-Zidocapt, Acezide, Capozide, Capto-Co, Enalapril/Hydchloroth, Innozide, Enalapril Mal, Innovace, Renitec, Enapren, Pralenal, Ednyt, Fosinopril Sod, Staril, Fositens, Fozitec, Lisinopril/Hydchloroth, Zestoretic, Carace Plus, Caralpha, Lisicostad, Lisinopril, Zestril, Carace, Acemin, Lisopress, Perindopril Erbumine, Coversyl, Perindopril Erbumine/Indapamide, Coversyl Plus, Quinapril HCL/Hydchloroth, Accuretic, Quinapril HCL, Accupro, Quinil, Ramipril, Tritace, Lopace, Ranace, Felodipine/Ramipril, Triapin, Trandolapril, Gopten, Odrik, Trandolapril/Verapamil HCL, Tarka, Imidapril HCL, Tanatril, Benazepril HCL, Cibacen, Perindopril Arginine, Coversyl Arginine, Perindopril Arginine/Indapamide, Coversyl Arganine Plus |
| **Other RAS drugs** | Aliskiren, rasilez |
| **Thiazides** | Bendroflumethiazide, Aprinox, Berkozide, Centyl, Neo-Naclex, Urizide, Neo-Bendromax, Chloroth, Saluric, Diuril, Chlotride, Chlortalidone, Hygroton, Cyclopenth, Navidrex, Hydchloroth, Esidrex, Hydrosaluric, Apo-Hyrdo, Hydroflumet, Hydrenox, Diucardin, Indapamide, Natrilix, Indaxa 2.5, Natramid, Opumide, Ethibide XL, Tensais XL, Varbim XL, Indipam XL, Mapemid, Rawel XL, Cardide SR, Alkapamid XL (HBS Healthcare Ltd), Alkapamid X (Rivopharm (UK) Ltd), Mefruside, Baycaron, Methylclothiazide, Enduron, Metolazone, Metenic, Xuret, Mykrox, Zaroxolyn, Xipamide, Diurexan |
| **Pot-Sparing Diuretics&Aldosterone Antag** | Amiloride HCL, Midamor, Berkamil, Amilispare, Amoride, Amilamont, Pot Canrenoate, Spiroctan-M, Soldactone, Spironol, Aldactone, Diatensec, Laractone, Spiretic, Spiroctan, Spirolone, Gx Spironol Abbolactone, Spirospare, Triamterene, Dytac, Eperenone, Inspra |
| **Potassium Sparing Diuretics & Compounds** | Amiloride HCL/Cyclopenth, Navispare, Co-Amilofruse, Frumil (Systemic), Lasoride, Fru-Co, Frusemek, Aridil, Froop Co, Komil, Co-Amilozide, Amil-Co, Hypertane 50, Moduretic, Normetic, Synuretic, Amilmaxco, Vasetic, Moduret 25, Delvas, Zida-Co, Amizide, Amiloride HCL + Loop Diuretic, Burinex-A, Co=Flumactone, Aldactide, Spiro-Co, Triamterene/Hydchloroth, Dyazide, Triam-Co, Triamaxco 50/25, Chloroth/Spironol, Spirool/Fureosemide, Frusens, Triamterene + Thiazides, Kalspare, Dytide |
| **Diuretics with potassium** | Bendroflumeth/Pot, Centyl-K, Neo-Naclex-K, Bumetanide/Pot, Burinek-K, Bumetanide/Amiloride HCL, Chlortalidone/Pot, Hygroton-K, Clopamide/Pot, Brinaldix-K, Cyclopenth/Pot, Navidrex-K, Cyclopenth/Amiloride HCL, Furosemide/Pot, Lasikal, Diumide-K Continus, Lasix + K, Hydchloroth/Pot, Esidrex-K, Hydrosaluric-K |
| **Beta-blockers** | Pindolol/Clopamide, Viskaldix, Sotalol HCL/Hydchloroth, Sotazide, Tolerzide, Timolol + Diuretic, Moducren, Prestim, Co-Tenidone, Tenoret, Tenoretic, Atenix Co, Techlor, Totaretic, Tertatolol, Artex, Celiprolol HCL, Celectol, Esmolol HCL, Brevibloc, Carvedilol, Eucardic, Gppe Tab, Monozide 10, Atenolol/Bendrofulmethiazide, Tenben, Penbutolol Sulf, Betapressin, Nebovolol, Nebilet, Hypoloc, Bisprolol Fumarate/Aspirin, Secadrex, Acebut HCl, Sectral, Practolol, Eraldin, Atenolol, Tenormin, Atenix, Antipressan, Vasaten, Totamol, Kentol, Atenamin, Gppe Cap, Kalten, Betaxolol HCl, Kerlone, Bisprolol Fumar, Monocor, Emcor, Isoten Mitis, Soprol, Cardicor, Bipranix, Concor, Soloc, Vivacor, Congescor, Labetalol HCl, Labrocol, Trandate, Metoprolol Fumar, Metoros, Metoprolol Tart, Betaloc, Lopressor, Mepranix, Arbralene, Toprol, Beloc Cor, Tensomex, Propanolol HCl + Diuretic, Inderetic, Inderex, Spiroprop, Propanolol HCl, Angilol, Apsolol, Bedranol, Berkolol, Inderal, Sloprolol, Lederpronol, Cardinol, Slo-Blok, Propanix, Beta-Prograne, Betadur, Avlocardyl, Probeta, Lopranol, Propatard, Slo-Pro, Syprol, Dupromex, Rapranol, Beprane, Atenolol/Nifedipine, Beta-Adalat, Tenif, Gppe Tab, Co-Betaloc, Lopresoretic |
| **Vasodilator antihypertensive drugs** | Diazoxide (Parent), Eudemine (Parent), Hydralazine HCl, Apresoline, Apo-Hydralazine, Minoxidil (Systemic), Loniten, Sod Nitroprusside |
| **Centrally acting antihypertensive** | Clonidine HCl (Vasodilator), Catapres, Methyldopa, Aldomet (Oral), Dopamet, Medomet, Lederdopa, Co-Caps Methyldopa, Metalpha, Methyldopa/Hydchloroth, Hydromet, Methyldopate HCl, Aldomet (Parent), Moxonide, Physiotens, Fisiotens, Normatens |
| **Alpha-Adrenoceptor Blocking Drugs** | Doxazosin Mesil (Hypertension), Cardura (Hypertension), Cascor (Hypertension), Doxadura (Hypertension), Slocinx, Colixil, Cardozin, Oxandosin, Doxzogen, Larbex, Raporsin, Indoramin (Hypertension), Baratol, Vadilex, Phenoxybenx HCl, Dibenyline, Phentolam Mesil, Rogitine, Prazosin HCl, Hypovase, Minipress, Kentovace, Alphavase, Teazosin HCl (Antihypertensive), Hytrin, Benph, Metirosine, Demser |
| **Calcium-Channel Blockers** | Amlodipine, Istin, Norvas, Norvasc, Astudal, Amlostin, Trimetazidine HCl, Vastarel, Diltiazem HCl, Tildiem, Calcicard, Britiazim, Angiozem, Adizem, Kentiazem, Dilaem, Slozem, Angitil, Calazem, Cardizem, Zemtar, Viazem, Dilcardia, Optil, Bi-Carzem, Zildil, Retalzem, Horizem, Zemret, Disogram, Tiamex, Kenzem, Uard, Diltiazem HCl/Hydchloroth, Adize-XL Plus, Felodipne, Plendil, Preslow, Cabren, Felotens, Felogen, Folpik XL, Keloc, Vacalpha, Parmid (Felodipine), Cardioplen, Neofel, Pinefeld, Isradipine, Presal, Lacidipine, Motens, Molap, Lercanidipine HCl, Zanidip, Nimodipine, Nimotop, Lidoflazine, Clinium, Nitrendipine, Baypress, Nicardipine HCl, Cardene, Nifedipine, Adalat, Adalat A.R, Vasad, Calcilat, Coracten, Angiopine, Adalate LP, Nifensar Xl, Kentipine, Cardilate, Nife-Wolff, Nifelease, Calanif, Hypolar, Adipine, Unipine, Nifedipin, Nimondrel, Slofedipine, Tensipine, Fortipine, Nifedotard, Genalat, Nifedipress, Nivaten, Coroday, Nifopress, Valni, Calchan, Neozipine, Adanif, Nidef, Prenlamine Lact, Synadrin, Verapamil HCl, Berkatens, Cordilox, Securon, Univer, Geangin, Verapress, Ethimil, Vertab, Zolvera, Ranvera, Vera-Til, Manidon, Perhexiline Mal, Pexid, Pexsig, Nisoldipine, Syscor, Sular, Mibefradil, Poisicor, Bufloedil HCl, Bufedil, Fonzylane, Valsartan/Amlodipine, Exforge |
| **Nitrates** | Isosorbide Dinit (Angina), Cedocard, Isoket, Sorbidilat, Isordil, Soni-Slo, Sorbichew, Sorbid, Sorbitrate, Vacardin, Imtack, Jeridin, Isocard, Angitak, Iso Mack, Carvasin, Isosorbide Mononit, Elantan, Modisal, Ismo, Monit, Mono-Cedocard, MCR 50, Imdur, Isotrate, Nu-Cross Isosorbide Mononit, Isin, Angitate, Tenkosorb, Astrodur MR, Pertil, Angeze, Dynamin, Isodur, Isotard, Monomax XL, Ketanodur, Monodur, Vaotrate, Monosorb, Chmydur, Zemon, Slomon, Carmil XL, Xismox, Cibral, Monigen, Trangina, Imo, Monomil, Ziotan, Phasonit, Relosorb, Eumon, Tardisc, Nyzamac SR, Isosorbide Mononit/Aspirin, Imazin |
| **Statins** | Atorvastatin, Lipitor, rosuvastatin, crestor, simvastatin, Zocor, fluvastatin, lescol, pravastatin, lipostat |

**Clinical Events**

| **Table S20. ICD10 codes for diagnoses considered in analyses** | | | |
| --- | --- | --- | --- |
| **Diagnosis** | **ICD10 Code** | **SNOMED CT** | **CTV3** |
| Hypertension | i10 | 1201005, 24184005, 38341003, 48146000, 56218007, 596218007, 59621000, 70272006, 78975002, 843821000000102, 843841000000109, 8458910000003, 8463710000003, 908631000000108, 908651000000101 | .G3.., .G31., .G36., .G3Z., G2…, G20.., G200., G201., G202., G203., G20z., G25.., G250., G26.., G28.., G2y.., G2z.., Gyu2., Xa0Cs, Xa3fQ, Xab9L, XaZbz, XaZWm, XaZWn, XaZzo, XE0Ub, XE0Ub, XE0Uc, XE0Ud, XE0W8, XM02V, XM09K, XM1C1, XSDSb |
| COVID-19 | u071, u072 | 1240581000000104, 1240751000000100, 1300721000000109, 1300731000000106 (EMISNQCO303 (Local EMIS code)) | Y22b7, Y22b8 |
| Diabetes | e10, e11, e13, e14 | 111552007, 11530004, 127012008, 127013003, 127014009, 190372001, 190388001, 190389009, 190416008, 190447002, 19429009, 201724008, 230577008, 237599002, 237602007, 237604008, 237632004, 237651005, 25093002, 268519009, 290002008, 310505005, 313435000, 313436004, 314771006, 314902007, 314904008, 335621000000101, 385041000000108, 395204000, 39710007, 401110002, 408540003, 420270002, 40279001, 420422005, 420436000, 420486006, 420514000, 420662003, 420715001, 420756003, 420789003, 421075007, 421326000, 421365002, 421468001, 421631007, 421750000, 421779007, 421893009, 421895002, 421896006, 422034002, 422088007, 422088007, 422099009, 422166005, 422228004, 422275004, 426875007, 43959009, 44054006, 443694000, 444073006, 46635009, 4855003, 49455004, 51002006, 5368009, 609561005, 609572000, 703136005, 703138006, 713702000, 713703005, 713706002, 71771000119100, 73211009, 739681000, 74627003, 8801005 | .C2.., .C21., .C22., .C2A., .C2C., .C2D., .C2E., .C2G., .C2G0, .C2G2, .C2G3, .C2Z., C10.., C1000, C1001, C102., C1041, C1051, C1060, C1061, C106y, C108., C1088, C1089, C108H, C109., C1097, C1098, C1099, C109C, C109E, C109F, C109H, C109J, C109K, C10B., C10C., C10D., C10E., C10E8, C10E9, CD10EH, C10EK, C10EL, C10EP, C10EQ, C10ER, C10F., C10F7, C10F8, C10F9, C10FC, C10FE, C10FF, C10FH, C10FJ, C10FK, C10FL, C10FM, C10FQ, C10FR, C10FS, C10G., C10J., C10N., C10P., C11y0, C1A0., Cyu2., X40J4, X40J5, X40J6, X40Ja, X40JA, X40Jb, X40JB, X40Jc, X40JC, X40Je, X40JE, X40JG, X40JI, X40JJ, X40JM, X40JO, X40JS, X40JY, X40JZ, Xaagd, XaCJ2, XaELQ, XaF05, XaFmA, XaFmL, XaFn7, XaFn9, XaIrf, XaIzM, XaIzN, XaIzQ, XaIzR, XaJQp. XaJSr, XaJUH, XaKyW, XaKyX, XaOPt, XaOPu, XE10E, XE10F, XE128, XE12A, XE12C, XE12M, XSETH, XSETK, XSETp |
| CHD* | i10, i11, i20, i21, i22, i23, i24, i25, i51 | 1201005, 129574000, 15990001, 1755008, 194802003, 194821006, 194823009, 194828000, 194841001, 194842008, 194843003, 194845005, 194849004, 194856005, 194857001, 22298006, 225566008, 233817007, 233819005, 233821000, 233823002, 233830008, 233838001, 233840006, 233843008, 233845001, 233847009, 233885007, 23687008, 24184005, 275511009, 275516004, 300995000, 304914007, 307140009, 311793000, 314116003, 315025001, 315026000, 315348000, 35928006, 36221001, 371068009, 38341003, 394659003, 398274000, 399211009, 401303003, 401314000, 413838009, 413844008, 414024009, 414545008, 414795007, 443502000, 4557003, 46109009, 48146000, 50570003, 52035003, 53741008, 54225002, 54329005, 56218007, 56265001, 57054005, 58612006, 59021001, 59621000,62695002, 64715009, 65340007, 65547006, 66189004, 67682002, 70211005, 7027006, 70422006, 719678003, 72092001, 732230001, 73795002, 76593002, 77970009, 78975002, 79009004, 810681000000101, 843821000000102, 843841000000109, 845891000000103, 846371000000103, 87343002, 90539001, 908631000000108, 908651000000101, 91335003 | .G3.., .G31., .G32., .G36., .G3Z., .G4.., .G41., .G42., .G420, .G43., .G44., .G440., .G441, .G443, .G444, .G445, .G445, .G45., .G451, .G452, .G45Z, .G46., .G47., .G48., .G49., .G4A., .G4Z., G2…, G20.., G200., G201., G202., G203., G20z., G21.., G211., G21z0, G25.., G250., G26.., G28.., G2y.., G2z.., G3…, G30.., G300., G301., G3011, G301z, G302., G303., G304., G305., G306., G307., G3070, G3071, G308., G309., G30A., G30X0, G30yz, G30z, G31.., G310., G311., G3110, G3111, G3112, G3113, G3114, G3115, G311Z, G312., G31y., G31y0, G31y3, G32.., G33.., G330., G3300, G331., G332., G33z., G33z3, G33z4, G33z6, G33z7, G33zz, G34.., G340., G3400, G3401, G341., G3410, G343., G34y0, G34y1, G34yz, G34z., G34z0, G35.., G37.., G38.., G384., G39.., G3y.., G3z.., G704., Gyu2., Gyu3., Gyu30, Ua1eH, X2006, X2007, X2008, X2009, X200a, X200A, X200B, X200c, X200C, X200e, X200E, X200G, X200H, X200I,X200J, X200K, X200L, X200M, X200N, X200O, X200P, X200Q, X200R, X200S, X200T, X200U, X200V, X200W, X200X, X200Y, X200Z, X202q, X202r, X202s, X203v, X776R, Xa0Cs, Xa0YL, Xa3fQ, Xa6Yx, Xa7nH, XaAC3, XaAZi, Xab9L, XaD2b, XaD2h, XaEgZ, xAeVd, XaFsG, XaFsH, XaG1Q, XaIf1, XaINF, XaIWM, XaIWY, XaYYq, XaZbz, XaXWm, XaZWn, XaZzo, XC0bX, XE0Ub, XE0Uc, XE0Ud, XE0Uh, XE0Ui, XE0Uj, XE0W8, XE0WC, XE0WE, XE0WG, XE2aA, XE2uV, XM02V, XM09K, XM0rN, XM1C1, XM1Qp, XSDSb, XSDT6 |
| Hypertensive heart disease* | i11 | 275516004, 36221001, 54225002, 56265001, 64715009, 77970009 | .G32., G21.., G211., G21z0, XE0Ue, XM1Qp |
| Ischemic heart disease* | i24, i25 | 1755008, 194821006, 194823009, 194823009, 194841001, 194842008, 194843003, 194845005, 194849004, 233817007, 233823002, 233840006, 275511009, 315026000, 315348000, 394659003, 399211009, 413838009, 413844008, 414545008, 443502000, 4557003, 46109009, 50570003, 53741008, 65340007, 66189004, 67682002, 719678003, 72092001, 732230001, 90539001 | .G42., .G420, .G43., G45., .G451, .G452, .G45Z, .G47., .G4Z., G3…, G31.., G310., G3110, G3115, G312., G31y., G31y0, G31y3, G32.., G34.., G340., G3400, G3401, G341., G3410, G343., G34y0, G34y1, G34yz, G34z., G34z0, G3y.., G3z.., G704., X2006, X200W, X200X, X200Y, X200Z, X776R, XaGYx, XaeVd, XaFsH, XaG1Q, XaINF, XC0bX, XE0Ui, XE0Uj, XE0WC, XE0WG, XE2aA, XM0rN |
| Angina* | i20 | 194828000, 22298006, 225566008, 233819005, 233821000, 233845001, 233845001, 23687008, 300995000, 314116003, 315025001, 35928006, 39827400, 413838009, 414024009, 414545008, 414795007, 443502000, 4557003, 53741008, 57054005, 59021001, 67682002, 810681000000101, 87343002 | .G4.., .G41., .G44., .G440, .G441, .G443, .G444, .G445, .G446, .G45., .G451, .G4A., G3…, .G311., G3111, G3112, G3113, G3114, G311z, G33.., G330., G3300, G331., G332., G33z., G33z3, G33z4, G33z6, G33z7, G33zz, G340., G37.., G39.., Gyu3., Gyu30, Ua1eH, X2007, X2008, X2009, X200A, X200B, X200c, X200C, Xa7nH, XaFsG, XaYYq, XE0Ui, XE0WA, XE0WE, XE2uV, XSDT6 |
| Myocardial Infarction* | i21, i22, i23 | 129574000, 15990001, 194802003, 194856005, 194857001, 22298006, 233830008, 233838001, 233843008, 233847009, 233885007, 304914007, 307140009, 311793000, 371068009, 398274000, 401303003, 401314000, 52035003, 54329005, 57054005, 58612006, 62695002, 65547006, 70211005, 70422006, 73795002, 76593002, 79009004, 91335003 | .G41., .G46., G48., G49., G30.., G300., G301., G3011, G301z, G302., G303., G304., G305., G306., G307., G3070, G3071, G308., G309., G30A., G30X0, G30yz, G30z., G35.., G38.., G384., X200a, x200e, X200E, X200G, X200H, X200I, X200J, X200K, X200L, X200M, X200N, X200O, X200P, X200Q, X200R, X200S, X200T, X200U, X200V, X202q, X202r, X202s, X203v, Xa0YL, XaAC3, XaAZi, XaD2b, XaD2h, XaEgZ, XaIf1, XaIWM, XaIWY, XE0Uh |
| Heart failure* | i50 | 10633002, 128404006, 195111005, 195112003, 195114002, 275514001, 360371003, 367363000, 389026000, 40541001, 42343007, 426611007, 446221000, 56675007, 71892000, 84114007, 85232009, 88805009, 92506005 | .G6A., .G6A1, .G6A2, .G6AZ, G58.., G580., G5801, G5802, G5803, G5804, G581., G5810, G583., G584., G58z., X102Y, X202l, XaEgY, XaO5n, XaWyi, XE0V8, XE0V9, XE0Wo, XE2QG |
| Pulmonary vascular disease* | i70, i73 | 195254008, 195295006, 195313002, 233956002, 233958001, 233959009, 233961000, 233962007, 234034005, 237897009, 25003006, 266259002, 266261006, 289923007, 300920004, 301755001, 302910002, 359557001, 371160000, 37151006, 38716007, 399957001, 400047006, 45281005, 50808002, 52403007, 58729003, 63491006, 6962006, 69742007, 72092001, 74725000, 77788005, 79256006, 81817003, 95691008 | .G6B4, .G8.., .G811, .G812, .G813, .G814, .G81Z, .G84., G85., G86., .G861, .G86Z, .G8Z., G342., G7…, G70.., G700., G701., G702., G7020, G703., G70y., G70y0, G70z., G73.., G730., G7300, G7301, G730z, G731., G7310, G733., G734., G735., G73y2, G73y6, G73y7, G73y8, G73yz, G73z,m G73z0, G73z1m G73zzm G7y.., G7z.., G9…, X203K, X203L, X203Q, X203R, X203S, X203T, X203U, X205U, X40R1, X50Ay, Xa0lV,Xa4eW, Xa7lT, Xa7lV, Xa84U, Xa9Br, XaB5d, XaB5V, XaB5W, XaBYF, XaE3G, XaJV4, XE0VN, XE0VO, XE0VP, XE0VQ, XE0VR, XE0X6, XE0X8, XE0XA, XE0XC, XM1Qu, XSDWB |
| Arrhythmia* | i44, i45, i47, i48, i49 | 12026006, 15964901000119107, 164947007, 16797001, 17338001, 17366009, 17869006, 195039008, 195042002, 195046004, 195060002, 195069001, 195070000, 195071001, 195072008, 195080001, 195083004, 195099005, 195101003, 195105007, 20143001, 204383001, 233892002, 233907003, 233910005, 233911009, 233916004, 233917008, 248629002, 251124007, 251150006, 251173003, 251175005, 251180001, 25569003, 270492004, 276796006, 27885002, 28189009, 29717002, 300996004, 30667004, 314208002, 33413000, 36083008, 361137007, 37760005, 418818005, 426749004, 427665004, 42807005, 440028005, 440059007, 44808001, 46935006, 49436004, 49710005, 4973001, 5370000, 54016002, 55475008, 59118001, 60423000, 62026008, 63467002, 63593006, 6374002, 6456007, 65778007, 66657009, 67198005, 698247007, 71792006, 717908006, 720448006, 74021003,74390002, 74615001, 86014007, 9651007 | .G65., .G651, .G652, .G653, .G654, .G655, .G656, .G657, .G65Z, .G66., .G67., .G670, .G68., .G681, .G682, .G68Z, .G69., .G691, .G692, .G69Z, G56.., G560., G561., G5611, G5612, G5613, G5614, G561z, G562., G5620, G562z, G564., G565., G5650, G5654, G5655, G565z, G566., G5660, G5662, G567., G5670, G5673, G5674, G56y., G56y0, G56y1, G56y4, G56y5, G56z., G56z0, G56zz, G57.., G570., G5700, G5701, G5792, G570z, G571., G572., G5720, G572z, G573., G5730, G5731, G5732, G5733, G5734, G5735, G5736, G573z, G574., G5740, G576., G5760, G5761, G5762, G5763, G5765, G56z, G577., G57y., G572, G57y3, G57y4, G57y6, G57y9, G57z., Gyu5a, P6y5., X2025, X2026, X2027, X2028, X2029, X202a, X202A, X202B, X202c, X202C, X202d, X202D, X202e, X202E, X202f, X202F, X202G, X202H, X202I, X202j, X202J, X202K. X202L, X202M, X202N, X202O, X202P, X202Q, X202R, X202S. X202T, X202U, X202V, X202W, X202X, X202Y, X202Z, X76JA, X76Je, X76Jf, X76Jg, X76Jh, X77A7, X77A8, X77A9, X77AA, X77Ab, X77AB, X77AC, X77AD, X77Ae, X77Af, X77AN, A77AW, X77AX, X77AY, X77Ba, X77Bb, X7BB, X77BC, X7BD, X77Be, X77BE, X77Bf, X77BF, X77Bg, X77Bi, X77BI, X77Bj, X77BJ, X77Bk, X77BK, X77Bl, X77Bm, X77Bn, X77Bo, X77BO, X77Bp, X77Bq, X77BQ, X77Br, X77Bs, X77BS, X77Bt, X77BT, X77BU, X77Bv, X77BV, X77Bw, X77BW, X77Bx, X77BX, X77By, X77Bz, X77BZ, X77C0, X77C1, X77C2, X77C3, X77C4, X77C5, X77C6, X77C7, X77C8, X77C9, X77CA, X908T, X908T, X90aM, X90C7, X90CA, X90fa, X90fz, Xa0D3, Xa0k6, Xa0Ky, Xa0lU, Xa0Ug, Xa0Ui, Xa0ZR, Xa0ZT, Xa0ZU, Xa0ZV, Xa0ZW, Xa2E8, Xa2jV, Xa7nI, Xa7nJ, Xa9Cn, XaaUH, XaBDJ, XaEga, XaOft, XaOgX, XE0V2, XE0V3, XE0V4, XE0V6, XE0V7, XE0Wc, XE0We, XE0Wg, XE0Wi, XE0Wk, XE0Wm, XE2QF, XM0PY |
| Stroke | i10 | 195206000, 195216008, 195217004, 20059004, 230690007, 230706003, 307756005 | .G7.., .G75., G66.., G667., G668., G669., X00D1, X00DI, X00DJ, X00DK, X00DR, X00DS, X00DT, Xa1hE, XaBE2, XaEGq, XE0X2, XE2aB |
| *CV comorbidity | | | |

**Clinical covariates**

| **Table S21. Read codes for clinical covariates and limits of spurious values** | | | | | | | |
| --- | --- | --- | --- | --- | --- | --- | --- |
| **Covariate** | **Read V2 Code** | **CTV3 Code** | **TPP Local code** | **SNOMED CT** | **Local EMIS Code** | **Lower limit*** | **Upper limit*** |
| **SBP^†^** | 2469 | 2469, XM02X | Y19e1 | 271649006, 163030003, 72313002 | EMISNQSY8 | 90mmHg | 280mmHg |
| **DBP**^‡^ | 246A | 246A, XM02Y | Y19e2 | 271650006, 1091811000000102, 163031004 | EMISNQDI86 | 30mmHg | 200mmHg |
| **BMI**^§^ | 22K | 22K | Y5627 | 60621009 | PCNQBO1 | 10kg/m^2^ | 100kg/m^2^ |
| *Values exceeding these limits were removed from the analyses  **^†^**Systolic blood pressure  ^‡^Diastolic blood pressure  ^§^Body mass index | | | | | | | |
